# PTGS2/GRP78 Activation Triggers Endoplasmic Reticulum Stress Leading to Lipid Metabolism Disruption and Cell Apoptosis, Exacerbating Damage in Bovine Mastitis

**DOI:** 10.3390/biom14121533

**Published:** 2024-11-29

**Authors:** Yan Chen, Bo Fang, Xian Liu, Wenkai Bai, Peiwen Liu, Zhiwei Duan, Ting Lu, Quanwei Zhang, Weitao Dong, Yong Zhang

**Affiliations:** 1 College of Veterinary Medicine, Gansu Agricultural University, Lanzhou 730070, China; xyzchenyan@163.com (Y.C.); fangb_ioi@163.com (B.F.); bwk20001129@163.com (W.B.); 18893134810@163.com (P.L.); 15002514846@163.com (Z.D.); asdlting@163.com (T.L.); 2 Gansu Key Laboratory of Animal Generational Physiology and Reproductive Regulation, Lanzhou 730070, China; zhangqw@gsau.edu.cn; 3 Lanzhou Centers for Disease Control and Prevention, Lanzhou 730030, China; lzcdcepi2010@126.com; 4 College of Life Sciences and Biotechnology, Gansu Agricultural University, Lanzhou 730030, China

**Keywords:** mastitis, lipoteichoic acid, endoplasmic reticulum stress, apoptosis

## Abstract

Lipoteichoic acid (LTA), an organic acid of Gram-positive bacteria, is closely related to mastitis in dairy cows. This study evaluates the effect of LTA-induced endoplasmic reticulum stress (ER stress) in vitro using MAC-T (mammary epithelial cells) and in dairy cows with mastitis. LTA stimulation significantly increases ER stress and apoptosis-related factors in MAC-T. Further analysis suggests that the increase in ER stress may be associated with interactions involving PTGS2 and GRP78. Protein structural studies indicate a strong interaction between PTGS2 and GRP78. Lipidomics results further demonstrate that LTA disrupts lipid balance in MAC-T cells, affecting lipid metabolism in the endoplasmic reticulum, including PC, PE, TAG, and DAG, thereby exacerbating inflammation and ER stress. In dairy cows with mastitis caused by Gram-positive bacterial infection, damaged epithelial cells, inflammatory cell infiltration, and apoptotic vesicles are observed in affected tissues. In contrast, tissues from healthy cows exhibit regular epithelial cells without inflammatory cells or apoptotic vesicles. Furthermore, a significant ER stress and apoptosis increase is observed in mastitis tissues. This study demonstrates the close association between LTA-induced cell damage and ER stress, contributing to understanding the mechanisms underlying LTA-induced damage and supporting strategies for mastitis prevention and control in dairy cows.

## 1. Introduction

In the dairy industry, mastitis is one of the most prevalent diseases in cows, leading to reduced milk yield and decreased milk quality, including lower levels of fatty acids in the milk [1,2]. Mastitis in cows is often caused by a combination of factors, including pathogenic microorganisms, intrinsic factors, and management practices. Among these, mastitis caused by Gram-positive bacteria has the highest incidence and the longest duration [3]. Of the various virulence factors of Gram-positive bacteria, the organic acid lipoteichoic acid (LTA) is particularly notable due to its complex interactions with host-pathogen relationships.

Lipoteichoic acid (LTA) is a major component of the cell wall in Gram-positive bacteria, characterized by its amphiphilic nature and its ability to anchor to the bacterial membrane. Structurally, LTA consists of a poly-glycerolphosphate backbone linked to glycolipids or lipoproteins, with variations in length and modification patterns depending on the bacterial strain [4]. Beyond its structural role, LTA plays crucial biological functions, including mediating bacterial invasion and activating the host’s innate immune response. Studies have shown that LTA can promote the maturation of IL-1β [5], activate IL-1β, IL-6, and TNF-α through the Toll-like receptor 2 (TLR2)/NF-κB signaling pathway, and upregulate the expression of prostaglandin-endoperoxide synthase 2 (PTGS2) [6], which further leads to cell apoptosis [7].

Continuous stimulation by *Staphylococcus aureus* (*S. aureus*) and its virulence factor LTA induces cellular stress responses, leading to excessive reactive oxygen species (ROS) and resulting in endoplasmic reticulum (ER) stress [7,8]. ER stress occurs when unfolded or misfolded proteins accumulate in the ER lumen, disrupting protein homeostasis and triggering the unfolded protein response (UPR). An upregulation of glucose-regulated protein 78 kDa (GRP78) is considered a key marker of ER stress [9]. GRP78’s critical functions include dissociating from and activating three UPR sensors: pancreatic ER kinase or PKR-like ER kinase (PERK), activating transcription factor 6 (ATF6), and inositol-requiring enzyme 1 (IRE1) [10]. The UPR aims to restore ER function. However, prolonged ER stress can shift from a protective response to triggering apoptotic signaling [11].

Recent studies have shown that LTA regulates the host cell endoplasmic reticulum (ER) stress response during *S. aureus* infection [12]. LTA can induce ER stress through various mechanisms, including direct interaction with TLR2 on the ER membrane or via downstream signaling pathways involving inflammatory cytokines and ROS [13]. Furthermore, LTA-mediated ER stress may exacerbate inflammation by amplifying cytokine production and activating inflammatory pathways, leading to tissue damage and disease progression [14].

To understand the mechanism by which LTA acted on mammary cells to further affect mammary tissue development after *S. aureus* infection in dairy cows, we attempted to explore the role of LTA in accelerating inflammation, further inducing endoplasmic reticulum stress, accelerating cell apoptosis, and ultimately leading to tissue damage and disease through transcriptome, untargeted lipidomics, and molecular biology methods. These studies also laid a foundation for the subsequent development of targeted prevention and control strategies.

## 2. Materials and Methods

### 2.1. Tissue Sample Preparation

Milk and mammary tissue were collected from dairy cows in Wuzhong City, Ningxia. Before collection, the somatic cell count (SCC) and pathogen isolation were used to assess the health status of dairy cows. According to the results of the SCC method and isolation, the cows were divided into a healthy cow group (NC group) and a clinical mastitis group (CM group), with 6 cows in each group. *S. aureus* was identified as the main pathogen in the CM group. Mammary tissue samples were fixed with 4% paraformaldehyde and 5% glutaraldehyde, respectively. Some samples were stored at −80 °C for future use.

### 2.2. Cell Culture and Immunofluorescence Staining

MAC-T (mammary epithelial cells) were donated by Associate Professor Longfei Xiao of the Beijing Academy of Agricultural Sciences. MAC-T were revived in a 37 °C water bath, and 10% fetal bovine serum (Invigentech, A6901FBS-500, Irvine, CA, USA) was added to DMEM/F12 medium (Hyclone, SH30022, Tauranga, New Zealand) and cultured in a 37 °C, 5% CO_2_ incubator. The cells were passaged with 0.25% trypsin-EDTA (Gibco, 25200-056, New York, NY, USA). MAC-T were placed in six-well plates, and when they reached about 70% confluence, they were treated with 0 or 10 μg/mL LTA (Sigma, L2515, St. Louis, MO, USA) for 24 h. Samples were collected and divided into a control group (CON group) and a 10 μg/mL LTA group (LTA10 group) [15].

The cells in the above two groups were washed with PBS to remove the supernatant, fixed with 4% paraformaldehyde at room temperature for 24 h, incubated with 0.3% Triton X-100 at room temperature for 5 min, and blocked with 5% BSA for 1 h. The primary antibodies PTGS2 (66351-1-Ig, proteintech, Wuhan, China), GRP78 (bsm-33224M, Bioss, Beijing, China), and CK18 (GB12232, Servicebio, Wuhan, China) were then incubated with a three-label four-color multiplex immunofluorescence kit (Aifang, AFIHC024, Changsha, China) at 37 °C in the dark for 1 h. After sealing with an anti-fluorescence quenching mounting medium, the expression level of cells was observed under a fluorescence microscope.

### 2.3. Transcriptome Analysis

In total, 1 × 10^7^ cells were collected and treated with 0 and 10 μg/mL LTA for 24 h, with three replicates per group. After total RNA was extracted with Trizol reagent, its quantity and purity were assessed using Bioanalyzer 2100 and RNA 6000 Nano LabChip Kit (5067-1511, Agilent, Santa Clara, CA, USA). mRNA was isolated from 1 μg of total RNA using Dynabeads Oligo (dT) (12310, Yeasen, Shanghai, China) and subjected to two purification steps. Subsequently, mRNA was fragmented at 94 °C for 10 min using a Magnesium RNA Fragmentation Module (E6150, NEB, Ipswich, MA, USA). Fragmented RNA was reverse transcribed into cDNA, and then the blunt end of each chain was A-tailed with an index adapter. The ligation product was purified and used for library quantification and quality detection. Gene Set Enrichment Analysis (GSEA) was used to sequence all genes from log2FC in descending order. Differentially expressed genes (DEGs) were identified using EdgeR, and then pathway enrichment analysis was performed using Gene Ontology (GO) and Kyoto Encyclopedia of Genomes (KEGG). KOBAS (http://bioinfo.org/kobasindex.php (accessed on 18 April 2024)). It is generally believed that pathway gene sets with |NES| > 1, *p* value < 0.05, and FDR/q-value < 0.25 are significant.

### 2.4. Untargeted Lipidomics Analysis

The collected cells were frozen and thawed in liquid nitrogen, sonicated on ice, added with the extract and sonicated on ice again, placed at −40 °C for 1 h, and the supernatant was vacuum-dried and then re-dissolved. After centrifugation, the supernatant was taken for detection. The target compounds were chromatographed on an Agilent 1290 ultra-high performance liquid chromatograph using a Phenomen Kinetex C18 liquid chromatography column. The liquid chromatography phase A was 40% water and 60% acetonitrile solution containing 10 mmol/L ammonium formate; the phase B was 10% acetonitrile, 90% isopropanol solution, and 50 mL of 10 mmol/L ammonium formate aqueous solution was added to every 1000 mL. A concentration time gradient elution was used. The sample tray temperature was controlled at 4 °C for injection, and the primary and secondary mass spectrometry data were collected under the following parameters of the Thermo Q Exactive Orbitrap mass spectrometer: Sheath gas flow rate: 30 Arb, Aux gas flow rate: 10 Arb, Capillary temperature: 320 °C (positive) or 300 °C (negative), Full ms resolution: 70,000, MS/MS resolution: 17,500, Spray Voltage: 5 kV (positive) or −4.5 kV (negative). *p* < 0.05, VIP > 2, and Log_2_FC were taken as significantly different lipids, and correlation and heat maps were drawn.

### 2.5. RNA Isolation, cDNA Synthesis and qPCR

All experiments were set up with 6 replicates, and the replicates were mixed and divided into three groups. Total RNA was extracted from bovine mammary glands/cells using the Trizol kit (Solarbio, Beijing, China) according to the manufacturer’s instructions. Subsequently, 1 μg of total RNA was reverse transcribed into cDNA using the Evo M-MLV RT Kit (Agbio, Changsha, China). Quantitative PCR (qPCR) was performed using 2× SYBR^®^ Green pro-Taq HS Premix (Selleck, Los Angeles, CA, USA) according to the manufacturer’s instructions, with β-actin as an internal control. qPCR primers were designed by NCBI (National Center for Biotechnology Information (https://www.nih.gov/ (accessed 9 January 2024)) and synthesized by Qingke Biological Technology Co., Ltd. (Xi’an, China). qPCR was performed using the LightCycler 96 Real-Time System (Roche, Basel, Switzerland).

### 2.6. Western Blot

The samples of dairy cow mastitis, which was mainly caused by *S. aureus* infection after bacterial identification, were selected. The sample pretreatment was the same as RNA extraction. IL-1β (66737-1-Ig, proteintech, Wuhan, China), IL-6 (21865-1-AP, proteintech, Wuhan, China), PERK (24390-1-AP, proteintech, Wuhan, China), ATF6 (24169-1-AP, proteintech, Wuhan, China), BAX (50599-2-IG, proteintech, Wuhan, China), Bcl-2 (60178-1-IG, proteintech, Wuhan, China), Caspase3 (bs-0081R, Bioss, Beijing, China), Caspase8 (bsm-33190M, Bioss, Beijing, China), PTGS2 (66351-1-Ig, proteintech, Wuhan, China), and GRP78 (bsm-33224M, Bioss, Beijing, China) proteins and MAC-T in breast tissues were measured by Western blot. Total proteins were extracted from mammary tissues/cells with cold RIPA buffer (Solarbio, Beijing, China) containing PMSF (Solarbio, Beijing, China). The primary antibodies were incubated overnight at 4 °C, followed by incubation with the corresponding secondary antibodies (goat anti-rabbit IgG secondary antibody, L3012, SAB, New York, NY, USA; goat anti-mouse IgG secondary antibody, L3032, SAB, New York, NY, USA) at 37 °C for 1 h. After band exposure, grayscale analysis of the bands was performed using Image J (Media Cybernetics Co., Rockville, New York, NY, USA).

### 2.7. ROS and Cell Apoptosis Assays

After 24 h of LTA treatment, the DCFH-DA probe (G1706, Servicebio, Wuhan, China) was diluted in DMEM/F12 medium and incubated in a CO_2_ incubator at 37 °C in the dark for 30 min. ROS levels were observed under an inverted fluorescence microscope. Cell apoptosis was detected using an FITC Annexin V Apoptosis Detection Kit I (556547, BD, Franklin Lakes, NJ, USA). Cells were trypsinized and resuspended. Annexin V-FITC and propidium iodide staining solution were added, followed by incubation at room temperature in the dark for 10–20 min, and then analyzed using a flow cytometer (CytoFlex, Beckman counter, Brea, CA, USA).

### 2.8. The Prediction of the Relationship Between PTGS2 and GRP78

The protein name was entered into the STRING website (https://cn.string-db.org/ (accessed on 16 February 2024)) to predict protein interactions. Predictions of protein secondary structure were obtained using SOPMA website (https://www.bing.com (accessed on 14 April 2024)). Receptor proteins were retrieved from the UniProt database. Water and ligands were removed from the receptor protein using PYMOL 2.3.4 software, and protein docking was performed using HDOCK website (http://hdock.phys.hust.edu.cn/ (accessed on 14 April 2024)). The protein with the highest score was selected for visualization analysis. The predicted structure was then analyzed using PYMOL v2.3.4 to find amino acid pairs that can interact with proteins to form hydrogen bonds. Finally, the predicted polymer structure was analyzed using PRODIGY website (https://play.prodigygame.com (accessed on 15 April 2024)).

### 2.9. H&E Staining and Morphological Observation

The fixed tissue sections were sequentially processed for H&E staining, including paraffin embedding, sectioning, deparaffinization, hematoxylin and eosin staining, differentiation, eosin counterstaining, dehydration, and mounting. Images were then acquired and analyzed using a Zeiss microscopy system. Fresh tissue samples for electron microscopy were the size of 1 mm × 1 mm × 3 mm and first fixed in 2.5% glutaraldehyde for 4 h at 4 °C. Subsequently, they were fixed with 1% OsO_4_ solution in 0.1 mol/L PBS for 2 h at room temperature and then dehydrated with a series of graded ethanol solutions. The dehydrated tissue was infiltrated, embedded, and polymerized in a 60 °C oven for 48 h, and the polymerized tissue blocks were cut into thin slices with 60 nm. The ultrastructure of the mammary gland was then examined using a transmission electron microscope (HT7700, HITACHI, Tokyo, Japan). The fixed tissue samples were dehydrated, dried, and gold-plated before being observed using a scanning electron microscope (Regulus 8100, HITACHI, Tokyo, Japan).

### 2.10. Statistical Analysis

All experimental data were analyzed using SPSS version 19.0 statistical software (SPSS, Chicago, IL, USA). Test data were expressed as “mean ± standard error of the mean”. One-way analysis of variance was used for comparison between the two groups. For comparison between multiple groups, a *t*-test followed by a least significant difference (LSD) post hoc test was used for pairwise comparison. When the probability value (*p*-value) was less than 0.01 (*p* < 0.01) or less than 0.05 (*p* < 0.05), it was considered statistically significant. Graphs were generated using GraphPad Prism 9.0 (GraphPad Software Inc., San Diego, CA, USA).

## 3. Results

### 3.1. Transcriptomic Profiling Reveals Mechanisms of LTA-Induced ER Stress Activation Leading to Apoptosis

**Figure 1:**
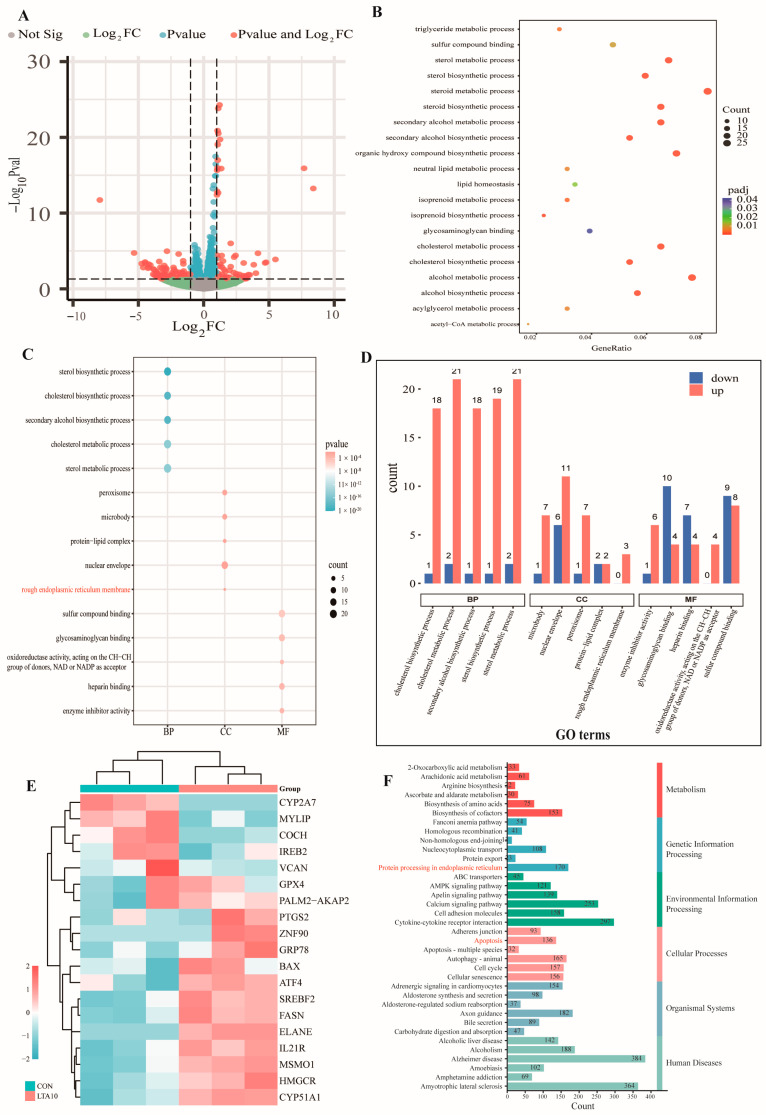
Transcriptomics reveals links between ER stress, steroid synthesis, and apoptosis in the LTA-induced MAC-T. (**A**) Volcano plot for the comparison between the CON group and the LTA10 group. (**B**) Scatter plot of enriched GO pathways statistics. (**C**) Dot plot for top 5 pathways of GO analysis. (**D**) Gene Ontology (GO) terms of the genes from the intersecting regions. (**E**) Heatmap of DEGs. (**F**) Classified KEGG pathways of the genes from the intersecting regions.

According to the results of SCC and bacterial isolation and identification, the bovine mammary tissue samples were divided into the NC group and the CM group. In addition, we found that Gram-positive bacteria are the main pathogens of bovine mastitis, so LTA was used to simulate the inflammation caused by Gram-positive bacteria. Before commencing cell experiments, the MAC-T inflammation model was constructed with LTA at a concentration of 10 μg/mL. The cells were subjected to transcriptome analysis, and a total of 20,345 genes were identified. A volcano map was constructed using all identified mRNAs (Figure 1A). All differentially expressed genes (DEGs) were screened, of which 191 were downregulated and 254 were upregulated. GO enrichment analysis of DEGs showed that DEGs were mainly related to lipid anabolism (Figure 1B). The top five pathways of BP, MF, and CC were taken for graphical drawing (Figure 1C). The corresponding DEGs and downregulated DEGs in these 15 pathways were counted (Figure 1D). The results showed that after LTA induction, DEGs were involved in biological processes such as lipid biosynthesis and endoplasmic reticulum membrane. Some differentially expressed genes were selected for the clustering heat map (Figure 1E), and the top six pathways in each type were selected for KEGG analysis and pathway mapping (Figure 1F). Among them, LTA was involved in protein processing, cell apoptosis, lipid synthesis, and metabolism. This suggests that LTA may affect lipid synthesis by causing cell inflammation, thereby affecting the cell endoplasmic reticulum process, generating ER stress, and eventually leading to cell apoptosis.

### 3.2. LTA Enhances ER Stress and Apoptosis in MAC-T

**Figure 2:**
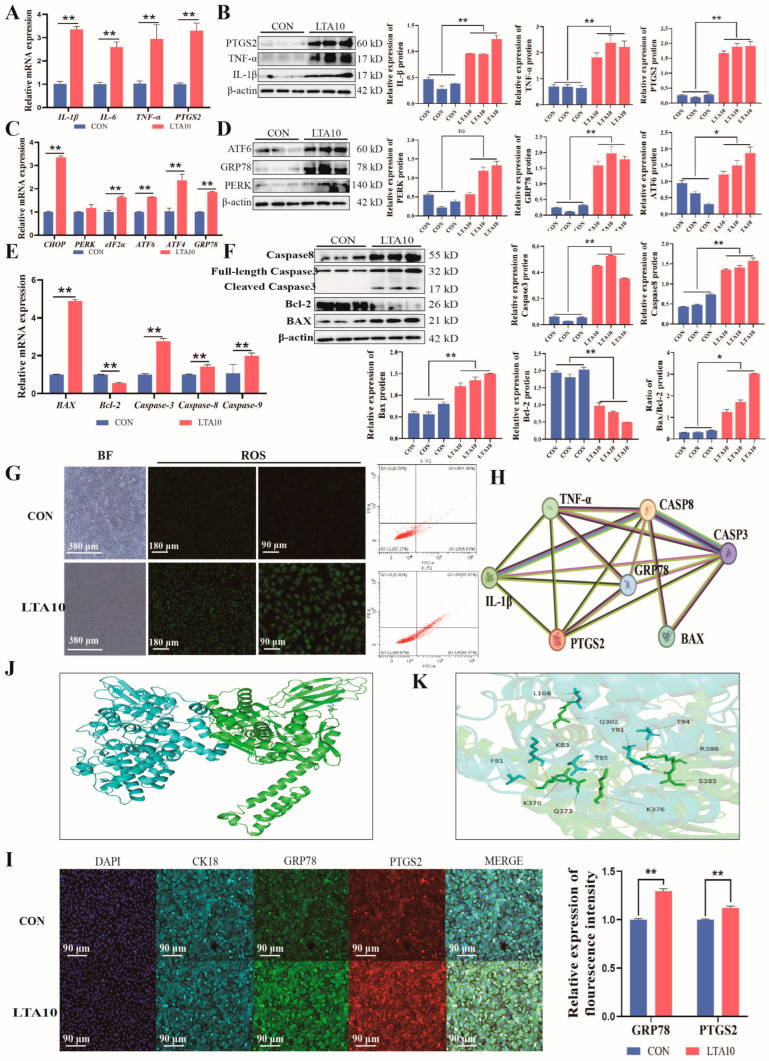
LTA aggravated inflammation, ER stress, and apoptosis in MAC-T. mRNA (**A**) and protein (**B**) relative expression of IL-1β, IL-6, TNF-α, PTGS2. mRNA (**C**) and protein (**D**) relative expression of CHOP, PERK, eIF2α, ATF6, ATF4, GRP78. mRNA (**E**) and protein (**F**) relative expression of BAX, Bcl-2, Caspase-3, Caspase-8, Caspase-9. (**G**) ROS assay and apoptosis assay in CON and LTA10 groups. (**H**) STRING predicted the relationship among IL1B, TNF, PTGS2, Caspase-3, Caspase-8, BAX, and GRP78. (**J**,**K**) HDOCK protein docking software performs PTGS2-GRP78 docking on proteins. (**I**) IF stain of CK18, PTGS2, GRP78. (* p < 0.05, ** p < 0.01, ns, not significant) (*n* = 3). Original images of (**B**,**D**,**F**) can be found in Supplementary Materials.

Previous studies have shown that 10 μg/mL of LTA can cause MAC-T cell inflammation. This part of the experiment focused on the expression of related factors in the CON and LTA10 groups (Figure 2). qPCR results showed that LTA could significantly increase the expression of cellular inflammatory factors *IL-1β*, *IL-6*, *TNF-α*, *PTGS2*, ER stress-related factors *CHOP*, *eIF2α*, *ATF6*, *ATF4*, *GRP78*, and apoptosis-related factors *BAX*, *Bcl-2*, *Caspase3*, *Caspase8*, *Caspase9*, but did not affect the expression of *PERK* (Figure 2A,C,E). Western blot data analysis results showed that the expression of IL-1β, IL-6, TNF-α, PTGS2, CHOP, eIF2α, ATF6, ATF4, GRP78, BAX, Bcl-2, Caspase3, Caspase8, and Caspase9 in the LTA10 group was significantly or very significantly higher than that in the CON group, while the expression of PERK was not significant, and the protein level was consistent with the gene level (Figure 2B,D,F). ROS detection showed that the staining signal in the LTA10 group was stronger than that in the CON group, indicating that the intracellular oxidative stress increased, and the ROS content increased, proving that the endoplasmic reticulum stress of the cells was aggravated. Flow cytometry results showed that the apoptosis in the LTA10 group was significantly more than that in the CON group (Figure 2G). In addition, the STRING protein interaction analysis of the above factors showed that the PTGS2 and GRP78 may interact to regulate ER stress (Figure 2H). The HDOCK molecular docking technology was further used to indirectly confirm the existence of a dockable hydrogen bond between PTGS2 and GRP78, with a binding energy of −13.7 kcal/mol (Figure 2J,K). Immunofluorescence co-localization results of PTGS2 and GRP78 showed that the levels of PTGS2 and GRP78 were significantly increased in the LTA10 group (Figure 2I). The localization results showed that PTGS2 and GRP78 were present in MAC-T at the same time, suggesting that they may be functionally related or similar in MAC-T.

### 3.3. Untargeted Lipidomics Show That LTA Disrupts Lipid Homeostasis and Exacerbates ER Stress

**Figure 3:**
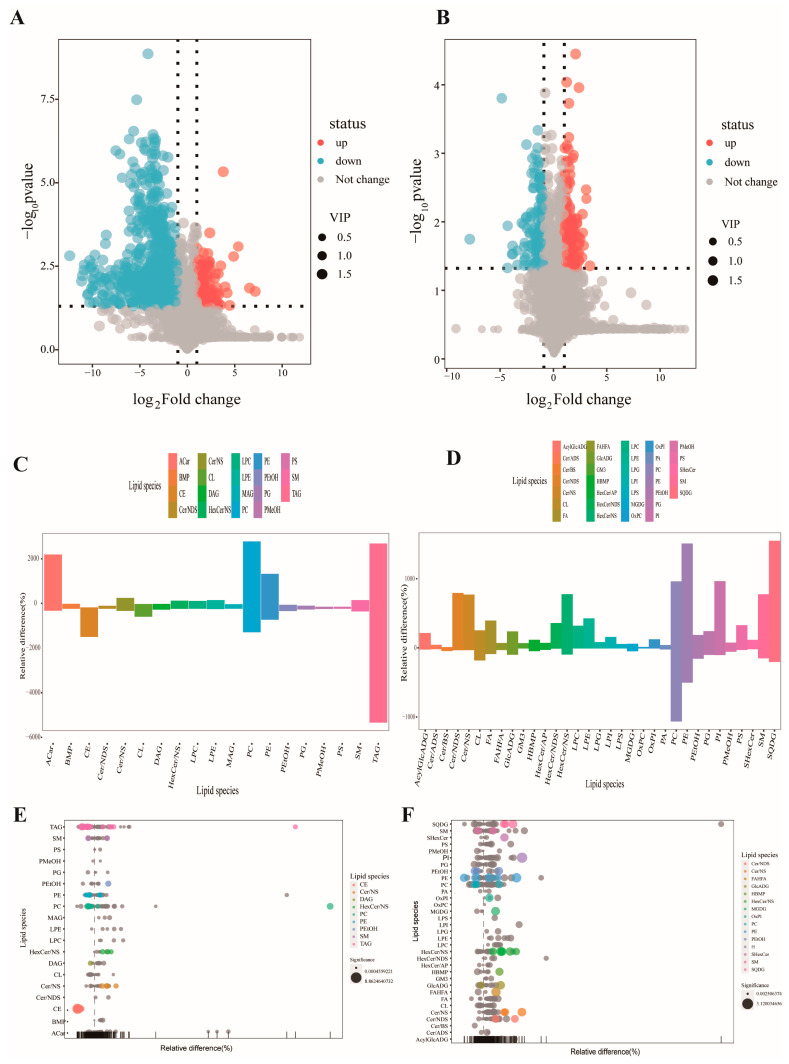
Untargeted lipidomics reveal that LTA enhances ER stress and disrupts lipid balance. (**A**) Volcano plot for all lipids in positive ionic mode. (**B**) Volcano plot for all lipids in negative ionic mode. (**C**) Bar-plot for differential lipids in positive mode. (**D**) Bar-plot for differential lipids in negative mode. (**E**) Bubble plot for differential lipids in positive mode. (**F**) Bubble plot for differential lipids in negative mode.

**Figure 4:**
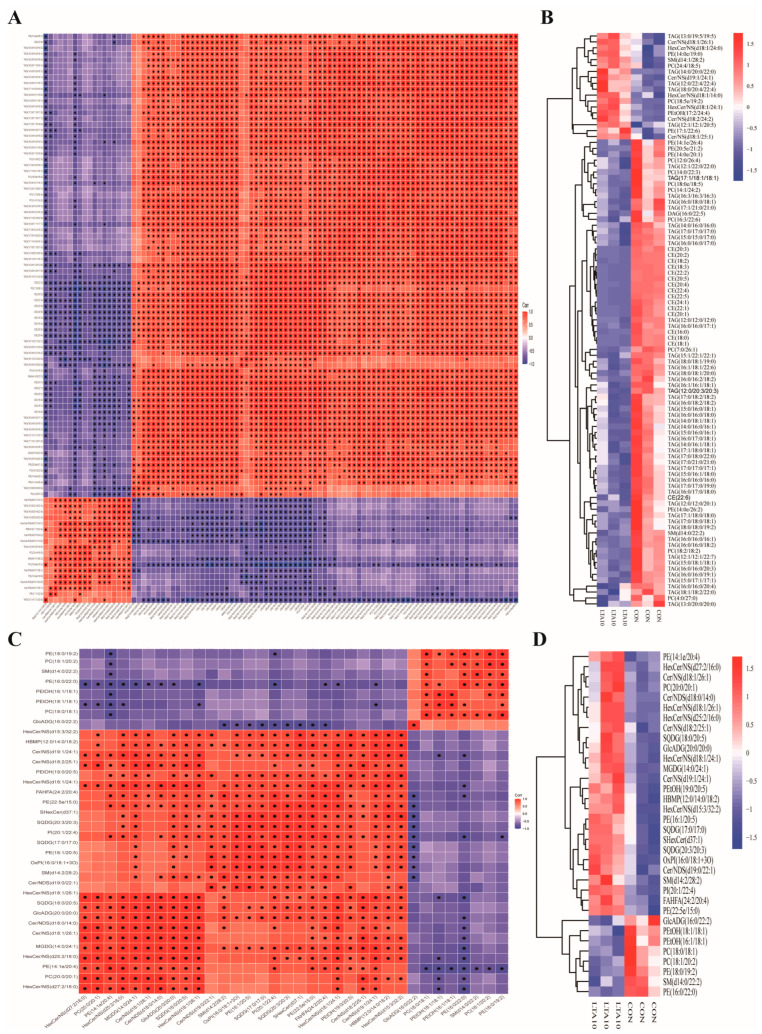
Untargeted lipidomics reveal that LTA enhances ER stress and disrupts lipid balance. (**A**) Correlation plot for differential lipids in positive mode. (**B**) Heatmap for differential lipids in positive mode. (**C**) Correlation plot for differential lipids in negative mode. (**D**) Heatmap for differential lipids in negative mode.

The occurrence of mastitis in dairy cows is usually accompanied by a decrease in milk production and quality. To study the changes in lipids in cows with mastitis, untargeted lipidomics analysis was performed on MAC-T. The results were analyzed using the statistical method of orthogonal partial least squares-discriminant analysis (OPLS-DA) (*p* < 0.05). Among them, 90 lipids were upregulated 549 lipids were downregulated in the POS mode (positive mode) (Figure 3A), 29 lipids were upregulated, and 35 lipids were downregulated in the NEG mode (negative mode) (Figure 3B). Bar graphs (Figure 3C,D) and bubble graphs (Figure 3E,F) were drawn for the degree of change and classification information of lipid content. The results showed that in the POS mode, LTA treatment increased the content of Acar, PC (phosphatidylcholine), and PE (phosphatidylethanolamine), and reduced the content of TAG (triacylglycerol) and CE (cholesteryl ester). In the NEG mode, the content of PE, Cer/NDS (ceramide non-hydroxyfatty acid-dihydrosphingosine), Cer/NS (ceramide non-hydroxyfatty acid-sphingosine), SQDG (sulfoquinovosyl diacylglycerol), etc., was increased. The enriched metabolites were correlated by Pearson calculation (Figure 4A,B). In the POS mode, PC (18:5e/19:2) was strongly positively correlated with PE (14:0e/19:2), and PC (24:4/18:5) and was strongly negatively correlated with TAG (16:0/16:0/20:3) and DAG (Diacylglycerol) (16;0/22:5). In the NEG mode, HexCer/NS (Hexosylceramide non-hydroxy fatty acid-sphingosine) (d27:2/16:0) was strongly correlated with PC (20:0/20:1) and PE (14:1e/20:4) and was strongly negatively correlated with PEtOH (Phosphatidylethanol) (18:1/18:1). In the cluster heat map (Figure 4C,D), the expression of PC (12:0/26:4), PC (14:0/22:3), PE (14:1e/26:4) in the POS mode and PC (18:1/20:2), PE (18:0/19:2) in the NEG mode in the LTA10 group were increased. Overall, the results suggest that LTA may be able to regulate lipid metabolism, thereby promoting the occurrence and development of inflammation.

### 3.4. Inflammation Promotes ER Stress in Clinical Mastitis Tissues

**Figure 5:**
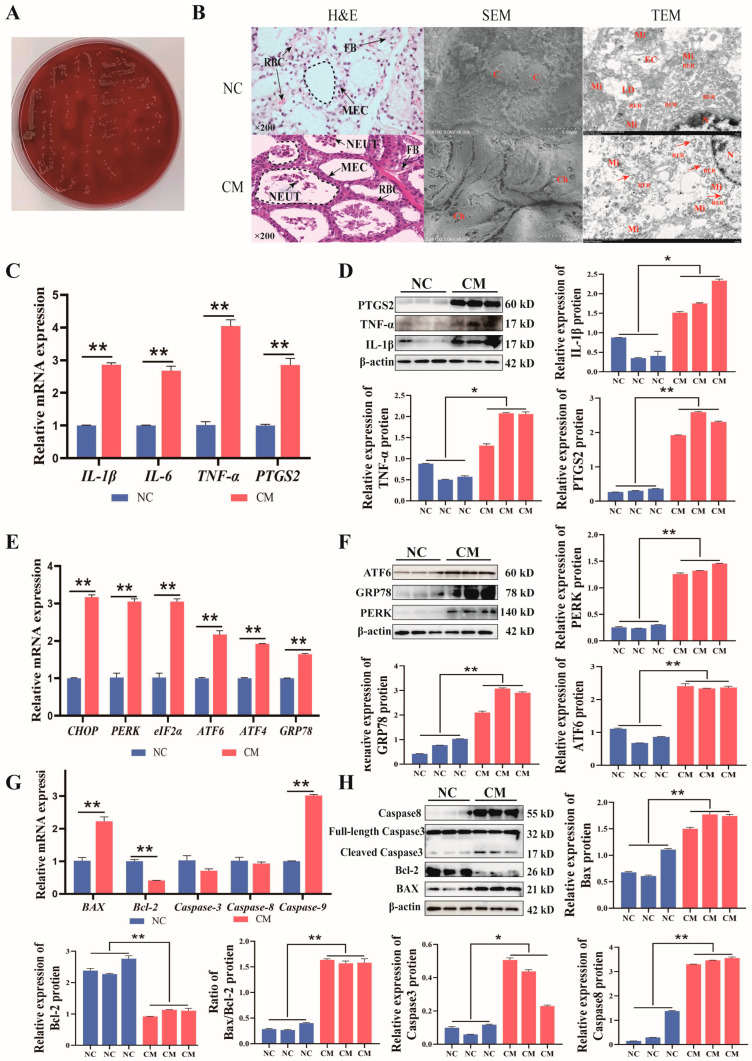
ER stress and apoptosis are highly expressed in clinical mastitis. (**A**) *S. aureus* isolated from clinical mastitis (**B**). Observations of H&E, scanning electron microscopy, and transmission electron microscopy in dairy cow mammary tissue. FB, fibroblast; MEC, mammary epithelial cells; RBC, red blood cell; NEUT, neutrophil in H.&E. C, epithelial cells, Ch, cell hole in SEM. N, the nucleus, M, mitochondria; RER, rough endoplasmic reticulum; LD, lipid droplets; EC, endocrine cells; red arrows indicate dilation, vacuolization, or rupture of ER cisternae in TEM. mRNA (**C**) and protein (**D**) relative expression of IL-1β, IL-6, TNF-α, PTGS2. mRNA (**E**) and protein (**F**) relative expression of CHOP, PERK, eIF2α, ATF6, ATF4, GRP78. mRNA (**G**) and protein (**H**) relative expression of BAX, Bcl-2, Caspase-3, Caspase-8, Caspase-9. (* p < 0.05, ** p < 0.01) (*n* = 3). Original images of (**D**,**F**,**H**) can be found in Supplementary Materials.

Histological analyses were performed to assess the damage caused by inflammation in the cows’ mammary tissue (Figure 5). The bacteria isolated from the cow’s milk showed hemolysis on the blood agar plate and were identified as *S. aureus* (Figure 5A). H&E staining showed that there was no inflammatory cell infiltration in the mammary tissue of cows in the NC group; the alveoli in the mammary tissue of cows in the CM group were atrophic and thin, some mammary epithelial cells were detached and damaged, and more inflammatory cells infiltrated in the alveoli. Scanning electron microscopy showed that the cell surface of the NC group was intact, while the cell surface of the CM group was damaged and had holes. Transmission electron microscopy results showed that compared with the NC group, the endoplasmic reticulum cisternae in the CM group were swollen and expanded, and the mitochondrial cristae were broken or missing. (Figure 5B). This showed that the mammary tissue of cows in the NC group was normal, while the mammary tissue of cows in the CM group had inflammation and apoptosis. The rough endoplasmic reticulum was observed to be swollen and ruptured, which is related to its biological function. qPCR results showed that inflammation can significantly increase *IL-1β*, *IL-6*, *TNF-α*, *PTGS2*, *CHOP*, *eIF2α*, *ATF6*, *ATF4*, *GRP78*, *PERK*, *BAX*, *Bcl-2*, *Caspase3*, *Caspase8*, and *Caspase9* (Figure 5C,E,G). Western blot data analysis results showed that the expression of IL-1β, IL-6, TNF-α, PTGS2, ATF6, PERK, GRP78, BAX, Bcl-2, Caspase3, and Caspase8 in the CM group was significantly or extremely significant compared with the NC group, and the protein level was consistent with the gene level, and the change trend was roughly consistent with that of MAC-T treated with LTA (Figure 5D,F,H). The above results show that 10 μg/mL LTA can form an inflammatory model of MAC-T. Due to inflammation, MAC-T ER stress and cell apoptosis increased, and changes in lipid content caused damage to the mammary tissue of dairy cows and reduced milk quality.

## 4. Discussion

**Figure 6:**
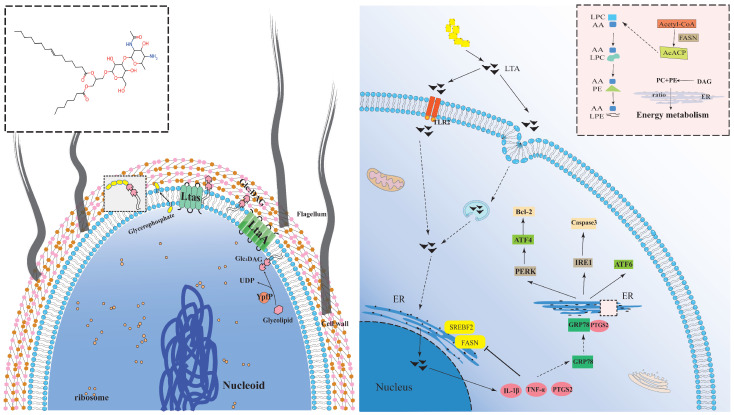
Model of LTA causes mammary gland injury in dairy cows. Analysis of the structure of LTA on the left side. LTAs are mainly composed of poly (GroP) chains, which are connected to the bacterial membrane via a glycolipid anchor. Glc_2_-DAG is synthesized by the YpfP enzyme and transferred to the outside of the membrane by the LtaA enzyme. Then, Glc_2_-DAG uses lipid phosphatidylglycerol (PG) as a substrate to repeatedly add GroP residues to the end of the growing chain through the LtaS enzyme to produce polyglycerol phosphate chains. The right picture shows that after LTA enters the cell, it invades the cell in the form of vesicles, passes through the ER to be processed in the nucleus, and triggers an inflammatory response, releasing the inflammatory factors IL-1β, TNF-α, and PTGS2. Inflammatory factors act on the endoplasmic reticulum, affecting the steroid synthesis pathway by reducing the expression of SREBF2 and FASN, and decreasing milk quality. At the same time, LTA-induced elevation of PTGS2 and GRP78 leads to ER stress and activates the expression of related factors such as PERK and ATF6, which trigger an apoptotic cascade with a decrease in Bcl-2 expression and an increase in caspase expression, leading to cell apoptosis.

Bovine mastitis is a common issue in dairy farming, and the resulting economic losses have garnered significant attention [16]. Previous studies have rarely explored the biological functions and mechanisms of LTA in mastitis. By integrating transcriptomic, metabolomic, and molecular biology results, we found that LTA-induced inflammation triggers ER stress, which lowers the expression of sterol regulatory element binding transcription factor 2 (SREBF2) and fatty acid synthase (FASN), affecting steroid production and subsequently reducing milk quality. Additionally, inflammation can cause damage through ER stress, leading to cell apoptosis. Based on these findings, we developed a mechanism diagram illustrating LTA-induced damage in bovine mammary epithelial cells (Figure 6). LTA, as an exogenous toxic factor, differs from various bacteria and can be recognized by TLR2 receptors on the cell membrane and enters the cell. Once inside the cell, it activates inflammation through the TLR2/NF-κB signaling pathway [17,18]. Under the combined stimulation of LTA and inflammation, the cell undergoes oxidative stress and ER stress. On one hand, ER stress affects steroid synthesis, and alters the inherent expression of PC and PE, leading to lipid dysregulation, which disrupts cellular energy metabolism and reduces milk yield and quality. On the other hand, it regulates the UPR response, promotes the expression of downstream apoptotic pathways, releases apoptotic signals, accelerates the apoptotic cascade, and causes cellular damage.

LTA is a major component of the cell wall in Gram-positive bacteria and is recognized by TLR2 on host cells. This recognition not only activates NF-κB but also mitogen-activated protein kinases (MAPK), leading to the expression and secretion of pro-inflammatory cytokines such as IL-1β, IL-6, and TNF-α [19]. These cytokines promote the recruitment of immune cells and enhance the inflammatory response within the mammary gland during *S. aureus* infection [20].

Additionally, inflammatory responses during immune reactions can lead to oxidative stress and the production of ROS, which can destroy the antioxidant balance of the organism. Low levels of ROS can serve as secondary messengers in cell signaling [21], while high concentrations of ROS can cause tissue damage. Persistent increases in ROS lead to ER stress, causing protein misfolding and ultimately resulting in cell death [22]. We examined ROS as a marker of ER stress, and the results showed that ER stress was increased in MAC-T upon stimulation with LTA, which is consistent with the literature. However, we did not perform testing at the tissue level because of sample collection issues. Apoptosis is a form of programmed cell death that plays a crucial role in maintaining organismal homeostasis. Studies have shown that ER stress can induce apoptosis and affect cellular function and structure [23]. Yu et al. discovered that ER stress can induce apoptosis in breast cancer cells through oxidative stress [24]. These findings confirm the interactions between ER stress, steroid metabolism, and inflammatory responses, ultimately leading to apoptosis. The UPR kinase PERK plays a key role in promoting apoptosis during ER stress [25,26]. PERK phosphorylates eIF2α, inhibiting protein translation while enhancing ATF4 translation [27]. Subsequently, ATF4 upregulates the transcription of the bZIP transcription factor CHOP [28]. In turn, CHOP downregulates the anti-apoptotic protein Bcl-2 and upregulates pro-apoptotic proteins BIM and PUMA, leading to apoptosis [29].

The complex interactions between these processes highlight the pathophysiological consequences of bacterial mastitis, including tissue damage and reduced milk yield. Bovine mastitis is a metabolic disease that affects energy metabolism, suggesting that lipid synthesis and metabolism are impacted during the progression of the disease [30]. Downregulation of the SREBF2 gene reduces the expression of the FASN gene in the LTA inflammation model, resulting in decreased fatty acid content in MAC-T-secreted milk. FASN activity controls apoptosis by fine-tuning mitochondrial thresholds [31]. Integrating transcriptomic and lipidomic data (Figure 3 and Figure 4), LTA was found to increase FASN expression while decreasing fatty acid levels in steroid metabolism, such as TAG (12:0/12:0/12:1) and PE (15:0/22:3), compared to the control group. Arachidonic acid (AA) is one of the most abundant and widely distributed polyunsaturated fatty acids in animals. In phagocytes and mast cells, free AA is typically bound to phospholipids, mainly PC [32]. During remodeling, AA is transferred from PC to PE [33]. In these two steps, LPC acts as an intermediary receptor, and LPE acts as a terminal receptor. In the endoplasmic reticulum, various membrane proteins convert DAG into PC and PE, and the PC/PE molar ratio within the cell can influence the energy metabolism of various organelles [34]. PC on the cell membrane has anti-inflammatory properties, inhibits lipid peroxidation, reduces neutral lipid content, scavenges peroxides, and prevents membrane damage by emulsifying and degrading lipids [35].

Notably, we have identified a novel link between inflammation and ER stress in MAC-T cells. Software predictions indicate that the binding free energy between PTGS2 and GRP78 is −13.7 kcal/mol, increasing the higher likelihood of interaction when the free energy is below −5 kcal/mol. This interaction was confirmed by immunofluorescence analysis of MAC-T cells (Figure 2). Interestingly, GRP78 and PTGS2 can act as acute regulators of steroidogenesis and inflammation [36]. Overexpression of GRP78 can rescue cells from oxLDL-induced stress, thereby restoring cellular homeostasis [37]. GRP78 targets the mitochondria, where it interacts with ADP/ATP carriers on the inner membrane and blocks ATP transport from the mitochondria to the cytoplasm, leading to apoptosis [38]. Elevated levels of IL-1β promote ferroptosis, while overexpression of GRP78 inhibits IL-1β-induced ferroptosis; silencing GPX4 diminishes this effect [39]. These effects are associated with PTGS2. Lipolysis products of blood triglycerides upregulate PTGS2 expression, inducing ER stress and the NF-κB pathway [40]. During the basic glandular characteristics in the front row, it is possible to pass through PTGS2 [41]. These findings suggest that bovine mastitis damage may be mediated through the cooperative regulation of lipid metabolism by these two genes, ultimately leading to cell apoptosis. However, current experiments are insufficient to fully support this conclusion, and we are conducting further studies to explore the specific mechanisms of their interaction.

## 5. Conclusions

LTA stimulation of MAC-T cells leads to oxidative stress, which in turn activates ER stress. This disrupts lipid balance, exacerbates inflammation, induces apoptosis, and consequently results in decreased milk quality, reduced yield, and cellular damage. Our findings suggest that LTA’s effects on these pathways may be primarily mediated through the induction of PTGS2 and GRP78 expression. Due to the limited number of experimental samples, the accuracy of some data may be affected; however, the data continue to have a high reference value. Future research should focus on elucidating the regulatory interactions between PTGS2 and GRP78, as well as the mechanisms by which PTGS2/GRP78 regulates apoptosis. These findings could further confirm that bovine mastitis severely impacts milk quality and causes additional harm to the organism. Additionally, they provide valuable support for the identification of target genes for the prevention and treatment of bovine mastitis.

## Data Availability

The data/models that support the study findings are available upon request.

## Supplementary Materials

The following supporting information can be downloaded at: https://www.mdpi.com/article/10.3390/biom14121533/s1, Original images of Figure 2B,D,F and Figure 5D,F,H can be found in Supplementary Materials.
