# Supplementary material for: PTGS2/GRP78 Activation Triggers Endoplasmic Reticulum Stress Leading to Lipid Metabolism Disruption and Cell Apoptosis, Exacerbating Damage in Bovine Mastitis
Source: Biomolecules. 2024 Nov 29;14(12):1533. doi: 10.3390/biom14121533 (PMC11673387; doi:10.3390/biom14121533)
Supplement: Supplementary file 1 [file biomolecules-14-01533-s001.zip › original image1.pptx]

## Slide 1
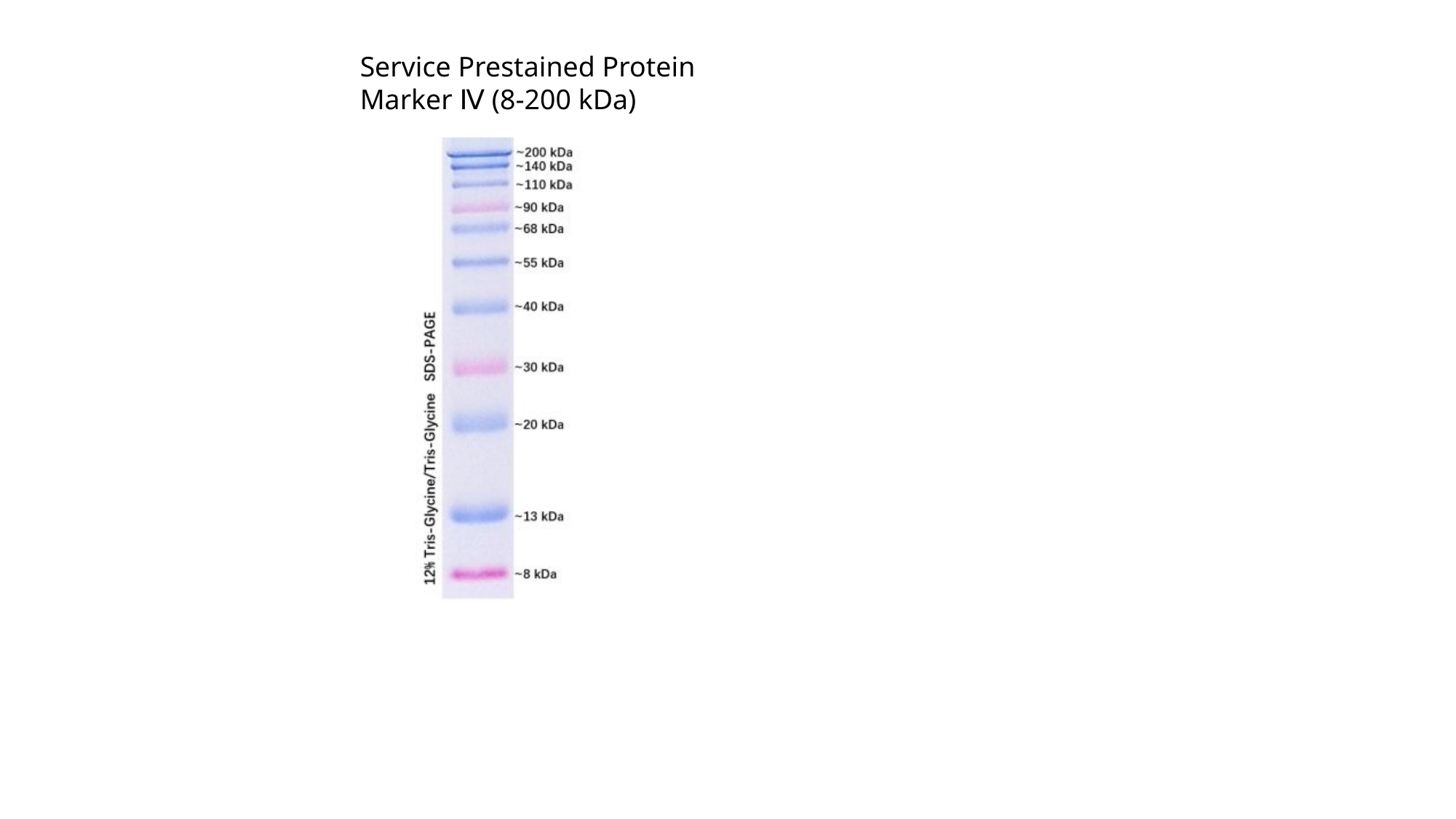

Service Prestained Protein Marker Ⅳ (8-200 kDa)

## Slide 2
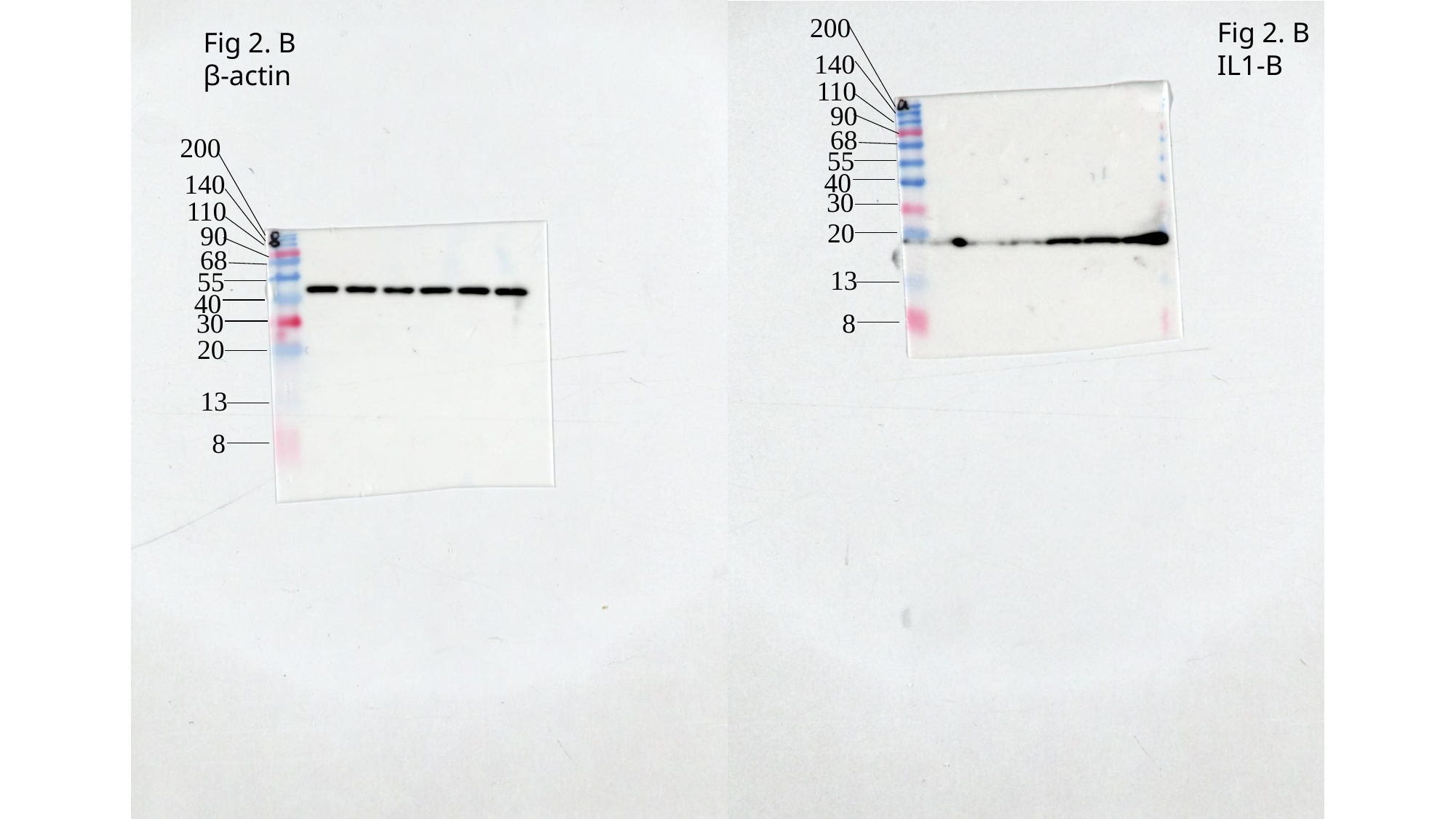

200
Fig 2. B
IL1-Β
Fig 2. B
β-actin
140
110
90
68
200
55
40
140
30
110
20
90
68
13
55
40
30
8
20
13
8

## Slide 3
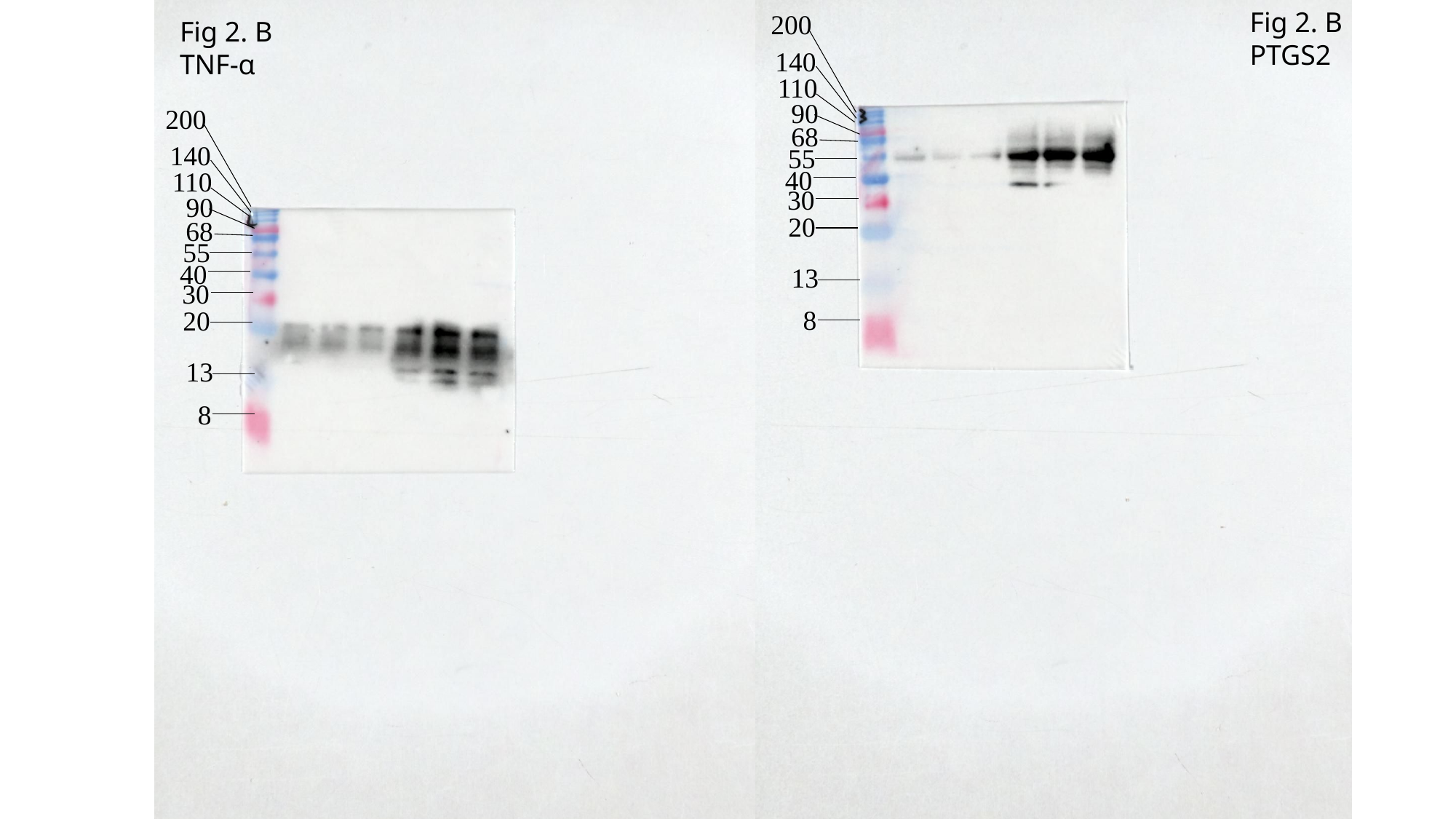

Fig 2. B
PTGS2
200
Fig 2. B
TNF-α
140
110
90
200
68
140
55
40
110
30
90
20
68
55
40
13
30
8
20
13
8

## Slide 4
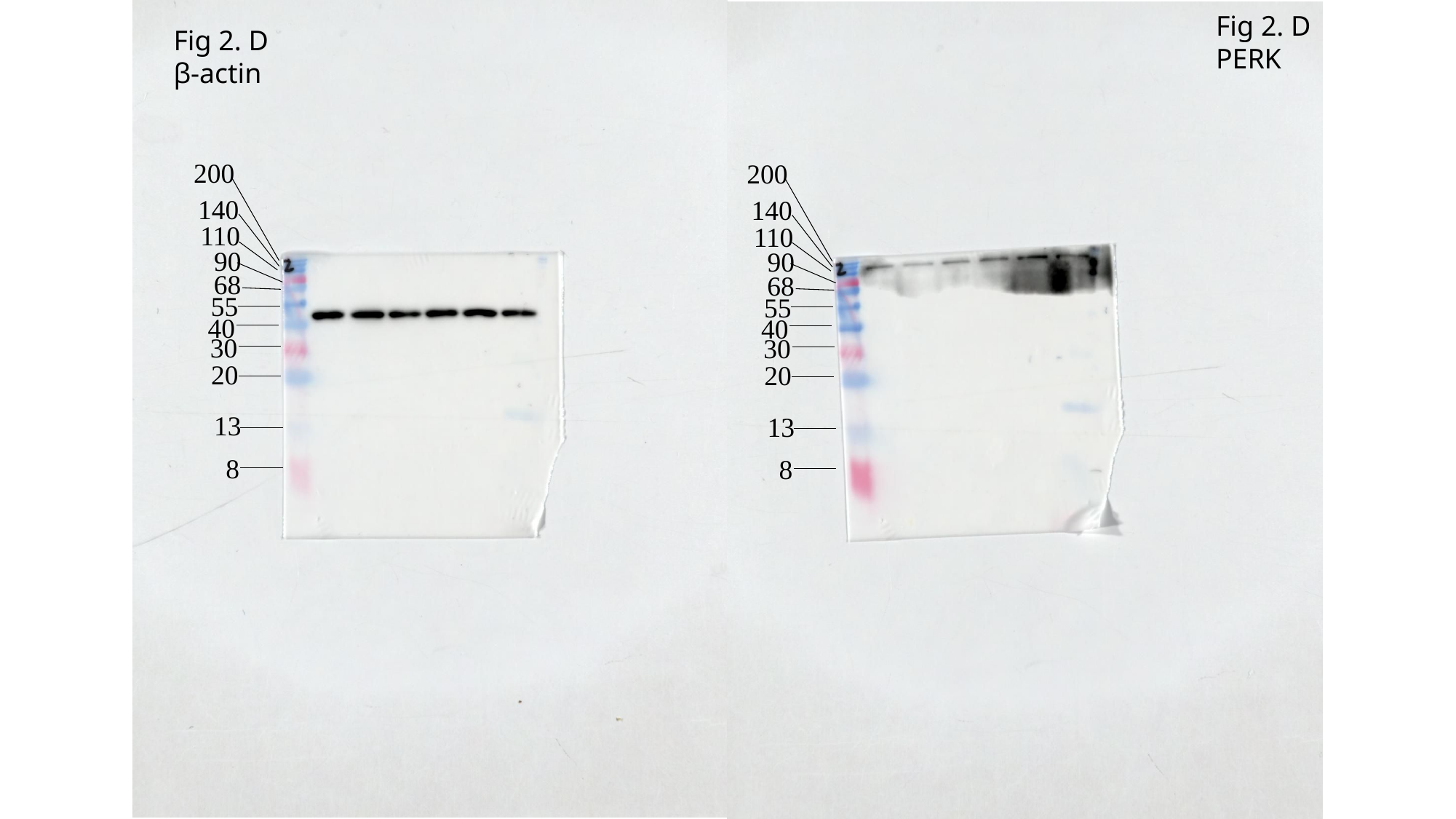

Fig 2. D
PERK
Fig 2. D
β-actin
200
200
140
140
110
110
90
90
68
68
55
55
40
40
30
30
20
20
13
13
8
8

## Slide 5
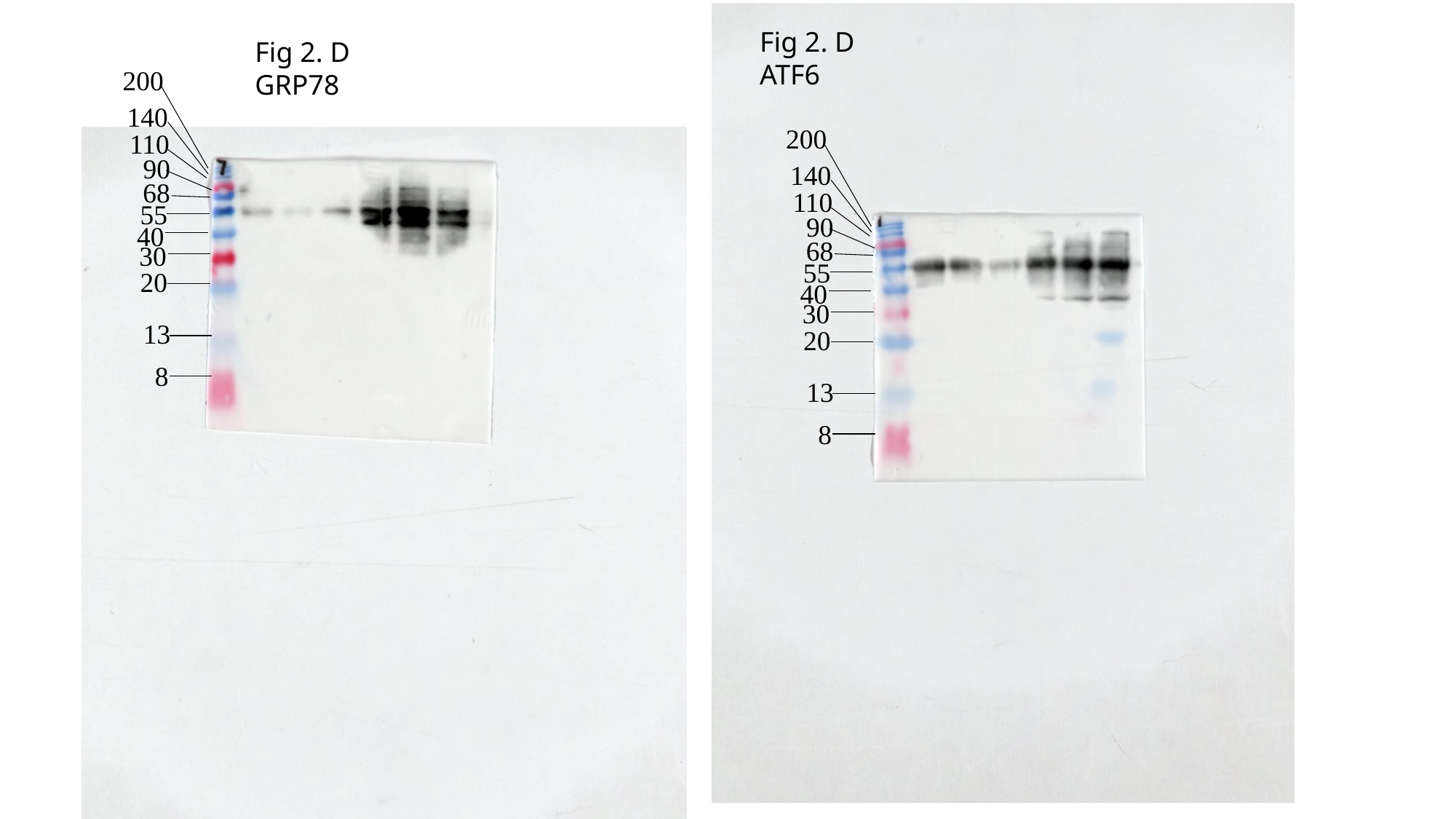

Fig 2. D
ATF6
Fig 2. D
GRP78
200
140
200
110
90
140
68
110
55
90
40
68
30
55
20
40
30
13
20
8
13
8

## Slide 6
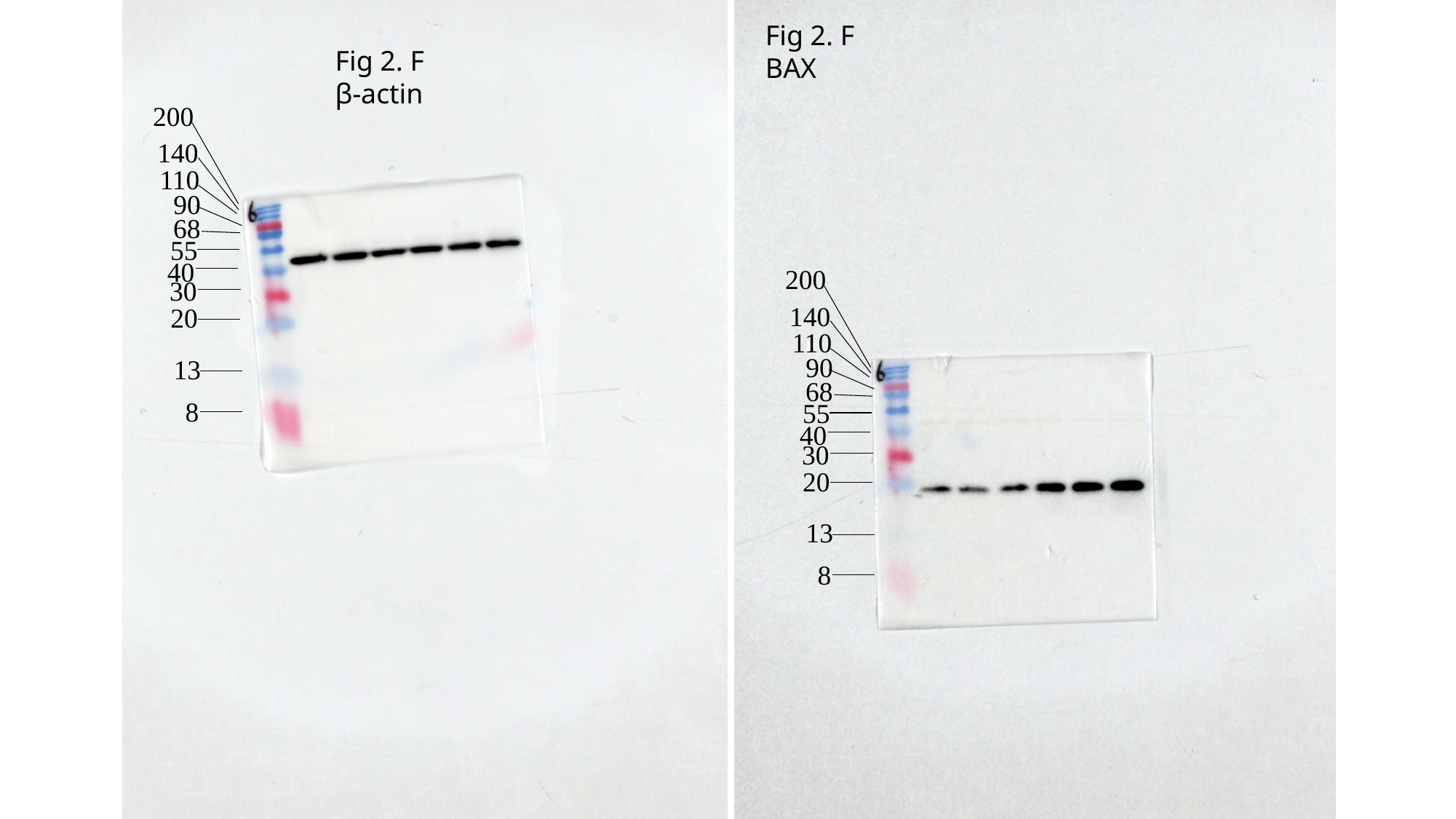

Fig 2. F
ΒAX
Fig 2. F
β-actin
200
140
110
90
68
55
40
200
30
140
20
110
90
13
68
8
55
40
30
20
13
8

## Slide 7
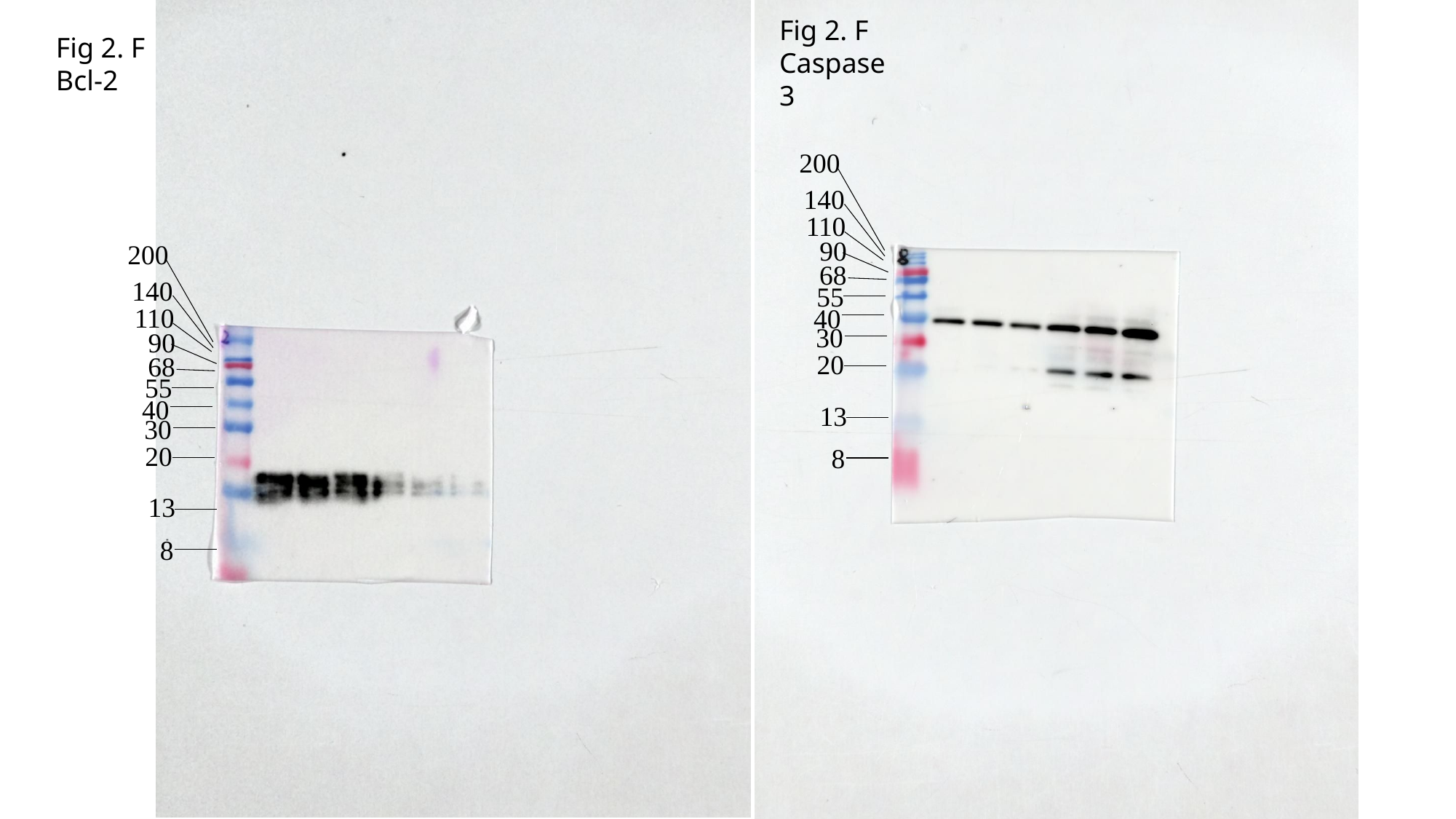

Fig 2. F
Caspase3
Fig 2. F
Βcl-2
200
140
110
90
200
68
140
55
110
40
30
90
20
68
55
40
13
30
20
8
13
8

## Slide 8
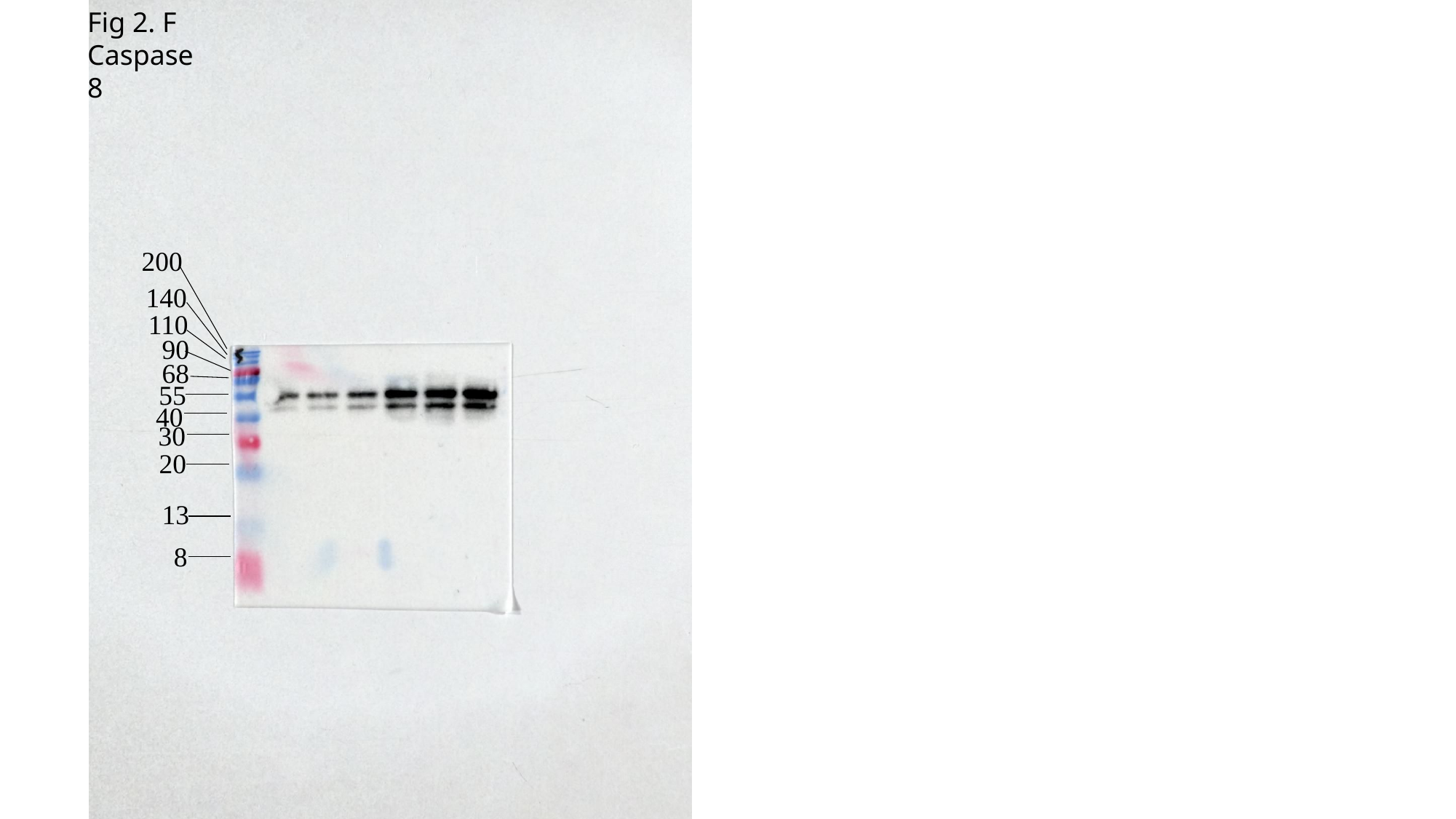

Fig 2. F
Caspase8
200
140
110
90
68
55
40
30
20
13
8

## Slide 9
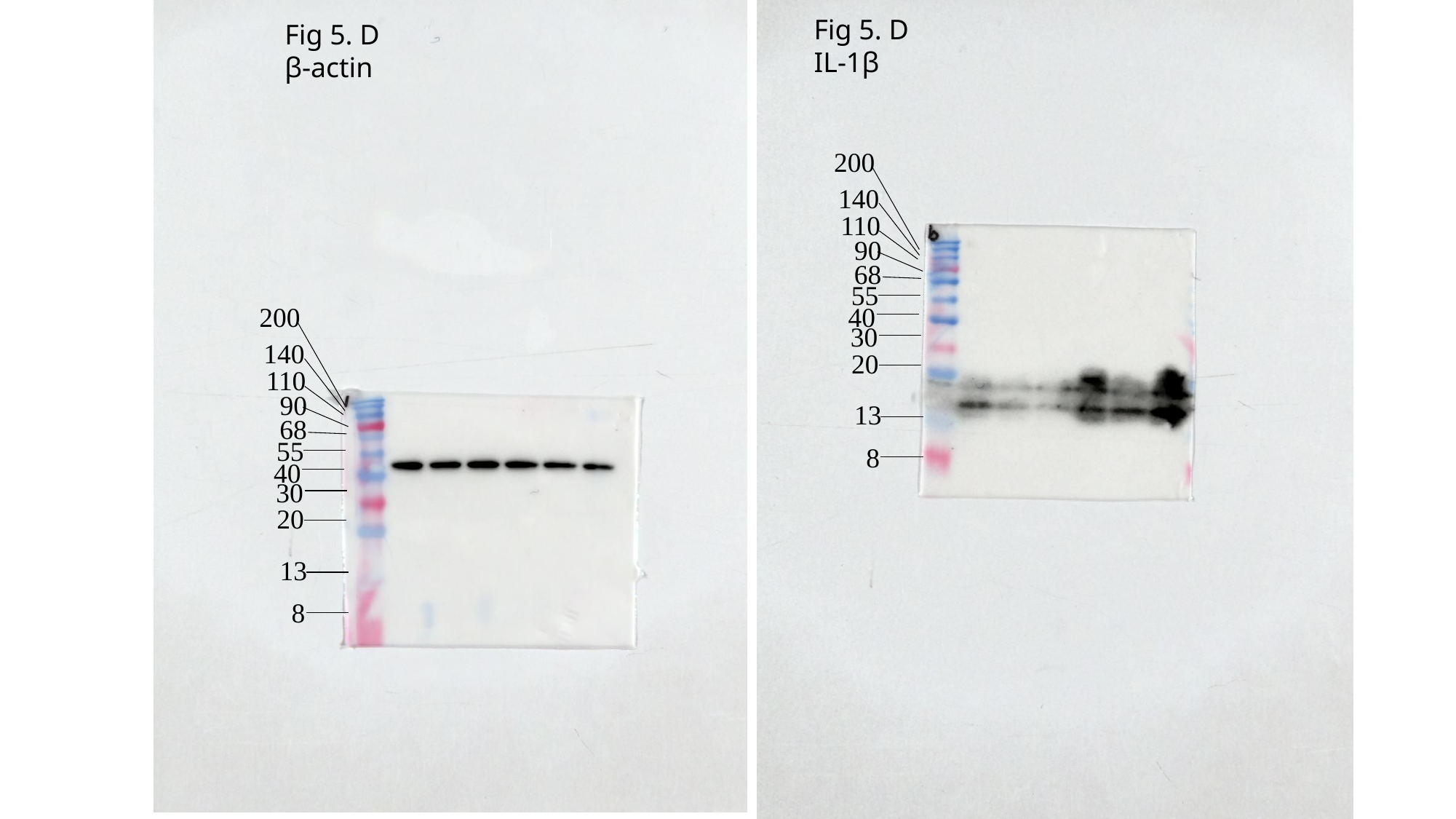

Fig 5. D
IL-1β
Fig 5. D
β-actin
200
140
110
90
68
55
200
40
30
140
20
110
90
13
68
55
8
40
30
20
13
8

## Slide 10
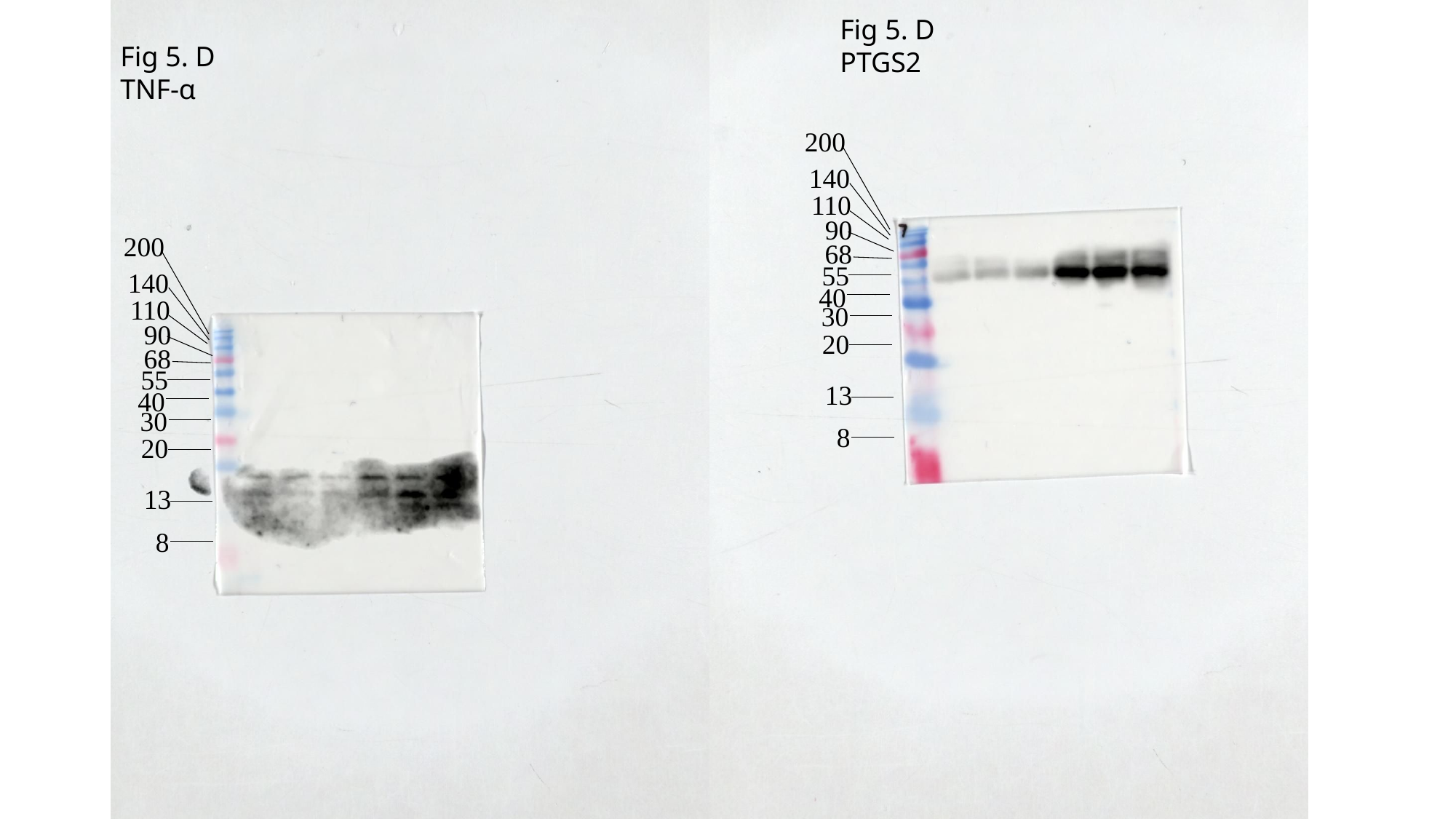

Fig 5. D
PTGS2
Fig 5. D
TNF-α
200
140
110
90
200
68
55
140
40
110
30
90
20
68
55
13
40
30
8
20
13
8

## Slide 11
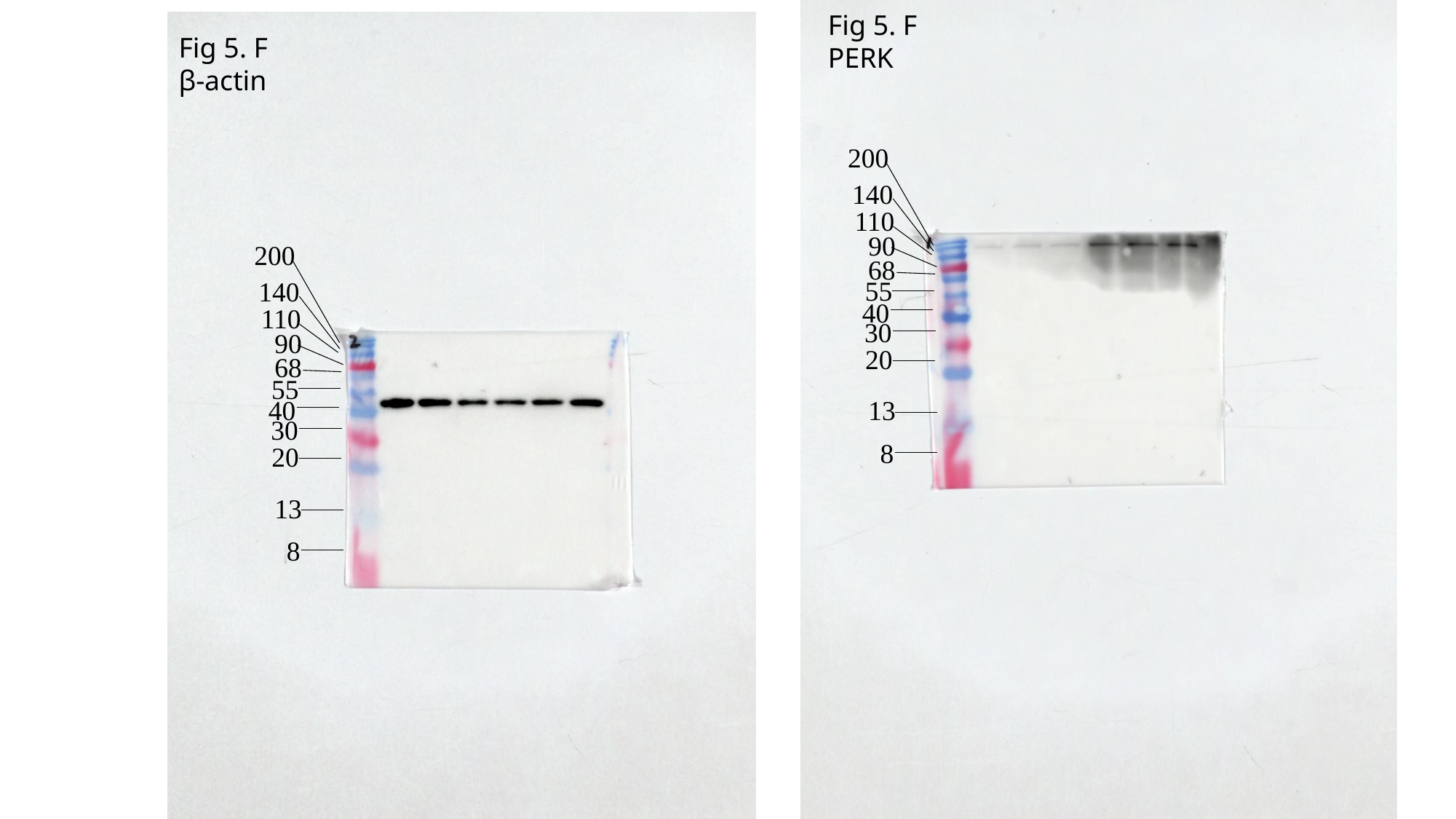

Fig 5. F
PERK
Fig 5. F
β-actin
200
140
110
90
200
68
55
140
40
110
30
90
20
68
55
13
40
30
8
20
13
8

## Slide 12
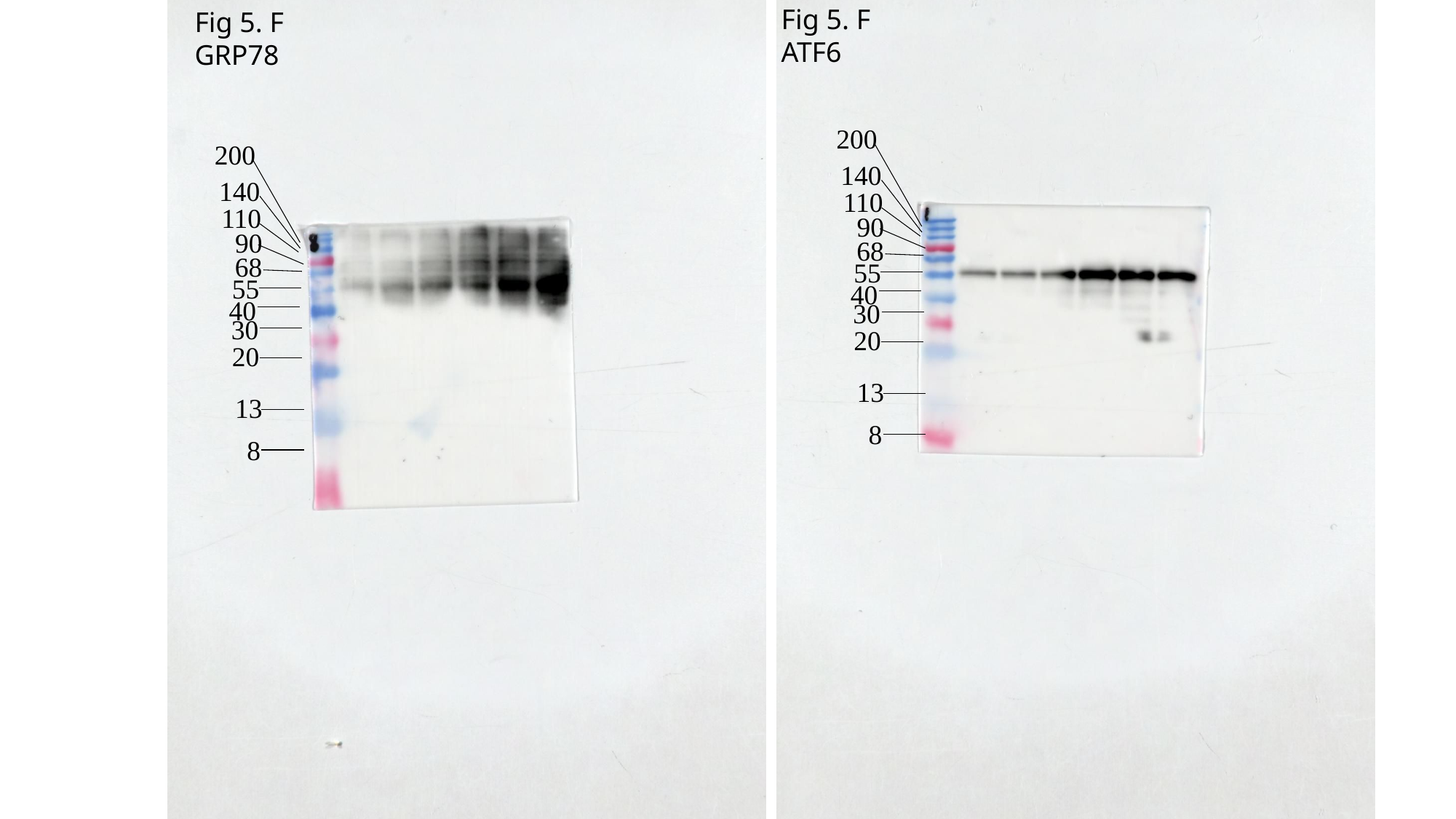

Fig 5. F
GRP78
Fig 5. F
ATF6
200
200
140
140
110
110
90
90
68
68
55
55
40
40
30
30
20
20
13
13
8
8

## Slide 13
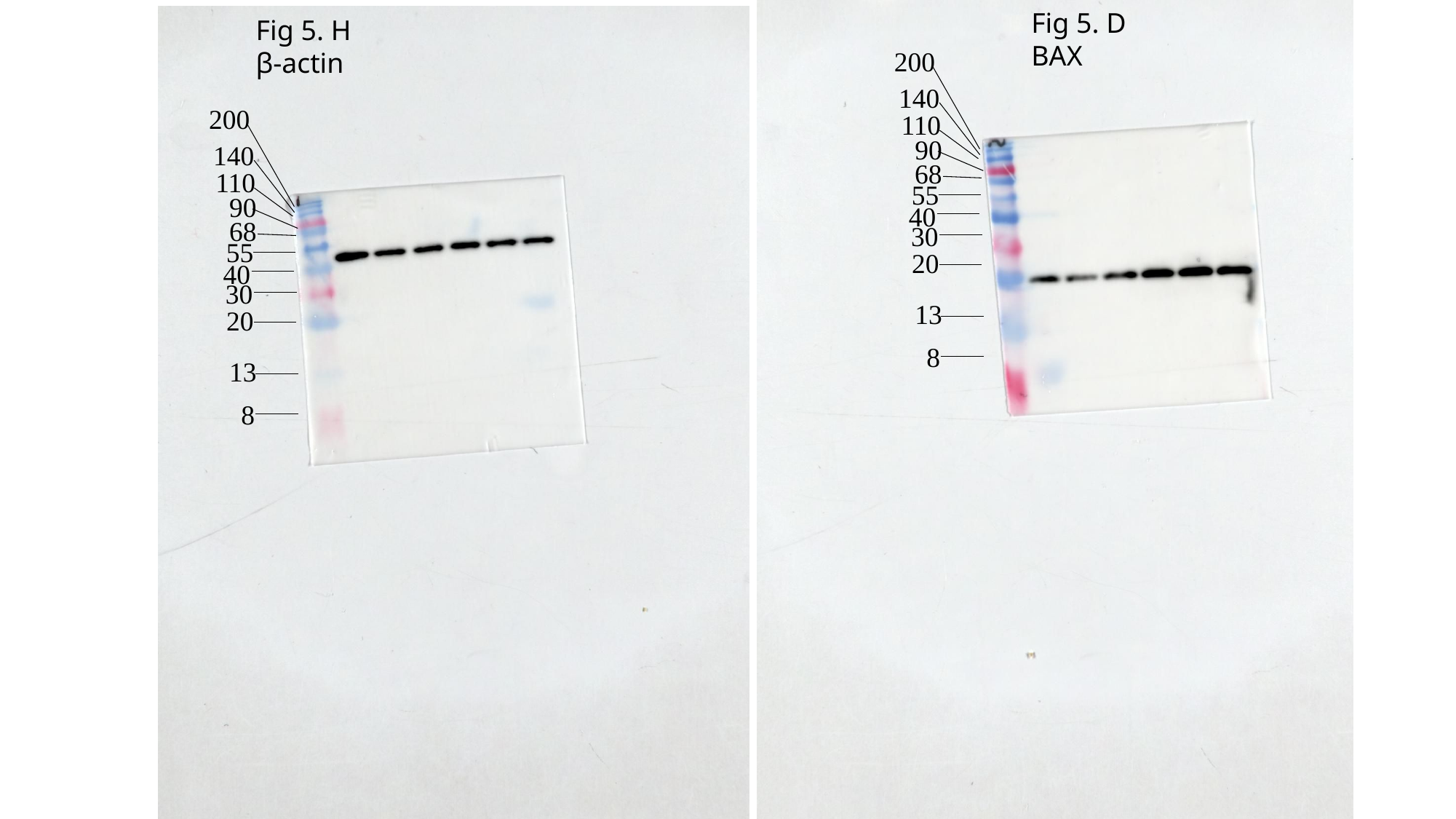

Fig 5. D
BAX
Fig 5. H
β-actin
200
140
200
110
90
140
68
110
55
90
40
68
30
55
20
40
30
13
20
8
13
8

## Slide 14
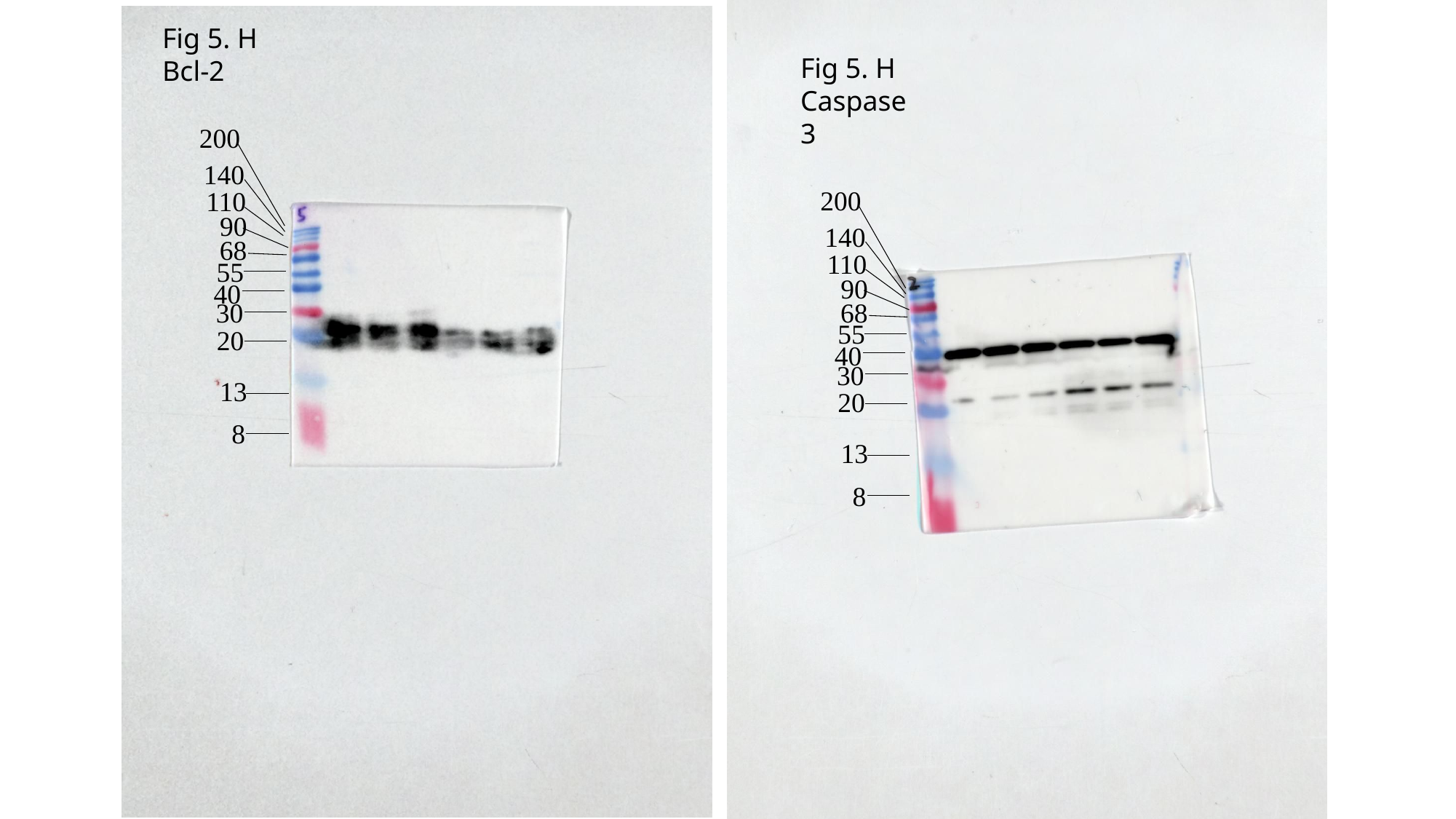

Fig 5. H
Bcl-2
Fig 5. H
Caspase3
200
140
200
110
90
140
68
110
55
90
40
68
30
55
20
40
30
13
20
8
13
8

## Slide 15
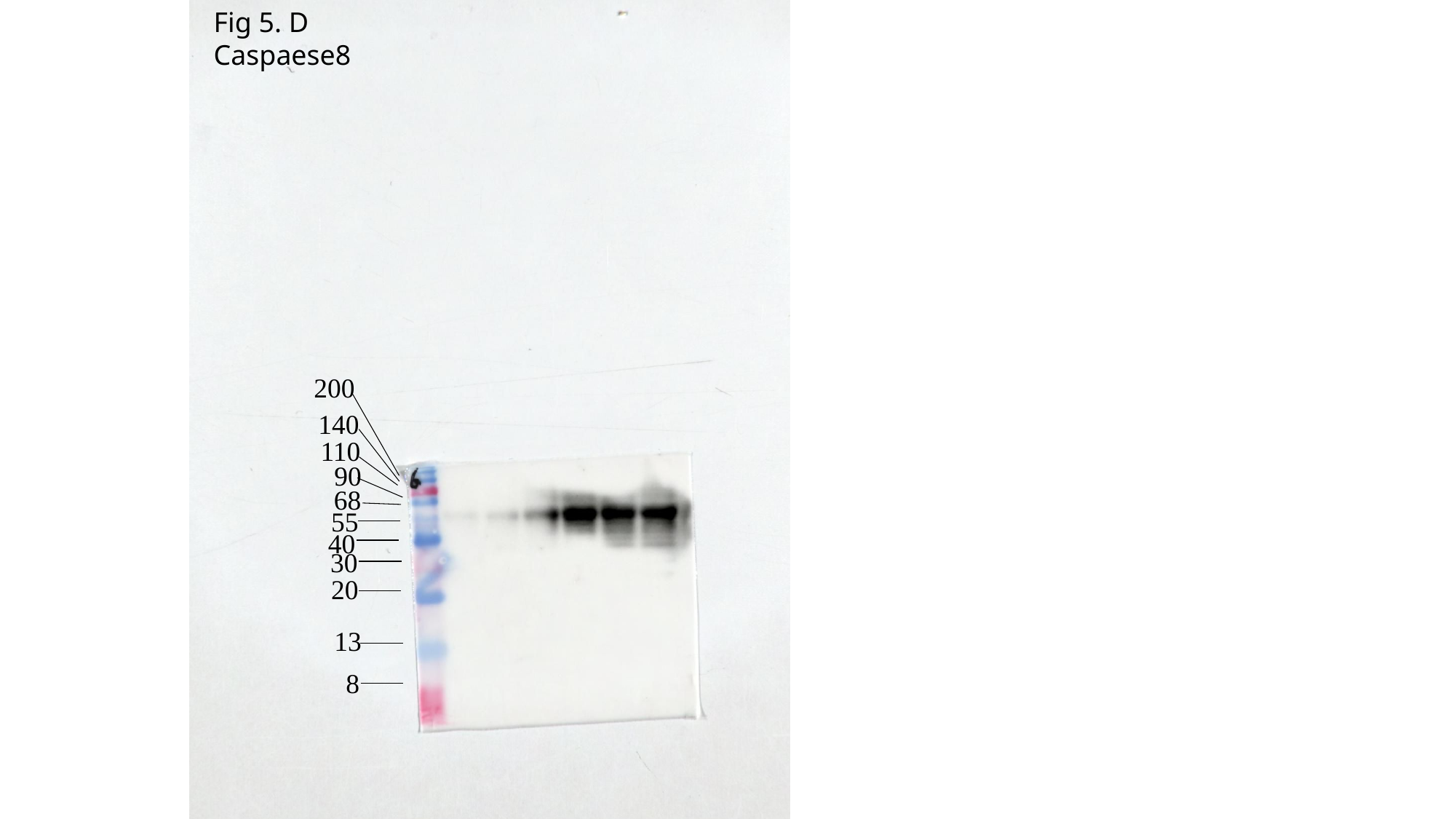

Fig 5. D
Caspaese8
200
140
110
90
68
55
40
30
20
13
8
