# Supplementary figures and images for: PTGS2/GRP78 Activation Triggers Endoplasmic Reticulum Stress Leading to Lipid Metabolism Disruption and Cell Apoptosis, Exacerbating Damage in Bovine Mastitis
Source: Biomolecules. 2024 Nov 29;14(12):1533. doi: 10.3390/biom14121533 (PMC11673387; doi:10.3390/biom14121533)

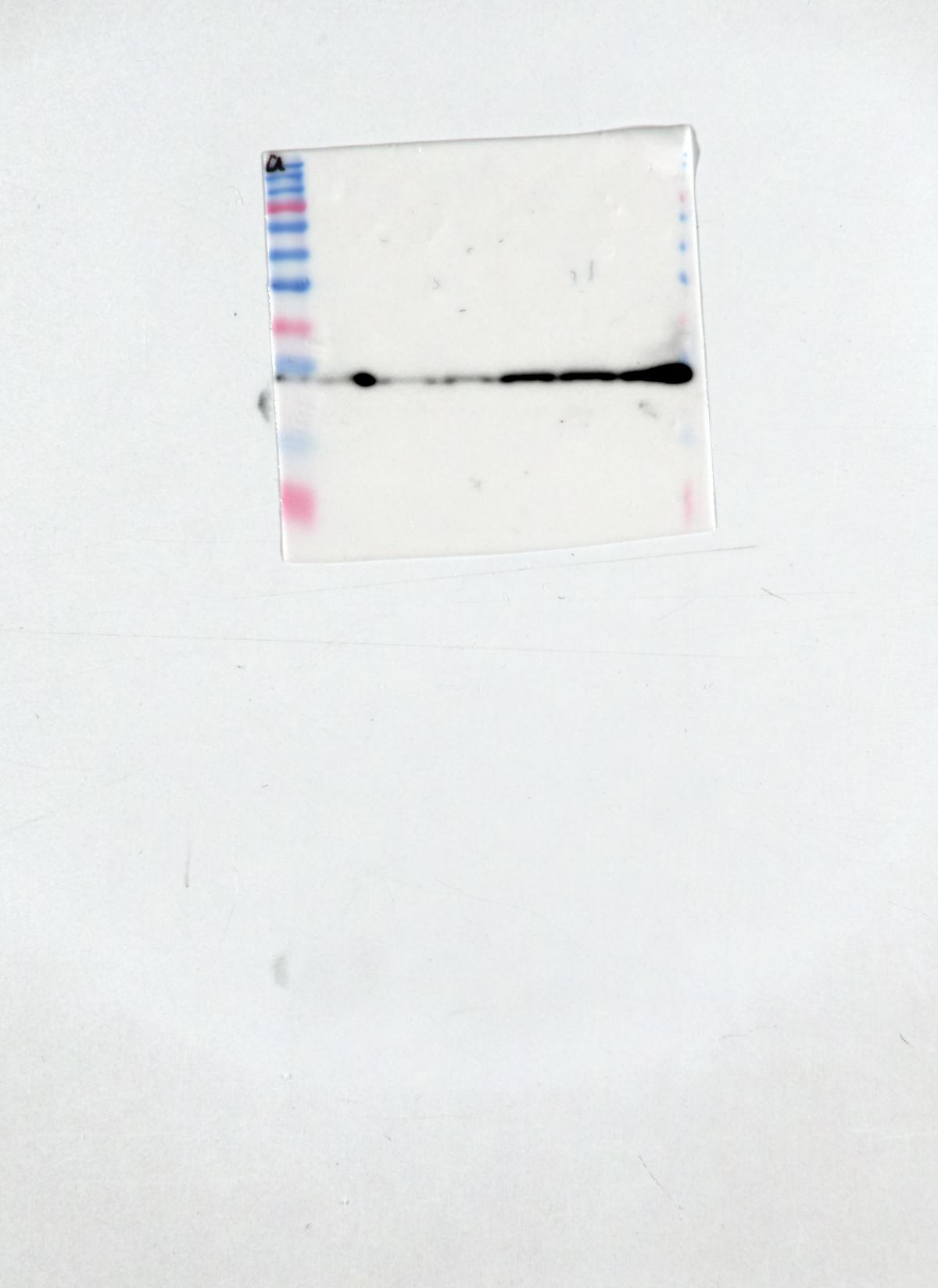

Supplement: Supplementary file 1 [file biomolecules-14-01533-s001.zip › Fig2.B IL-1a┬.jpg]

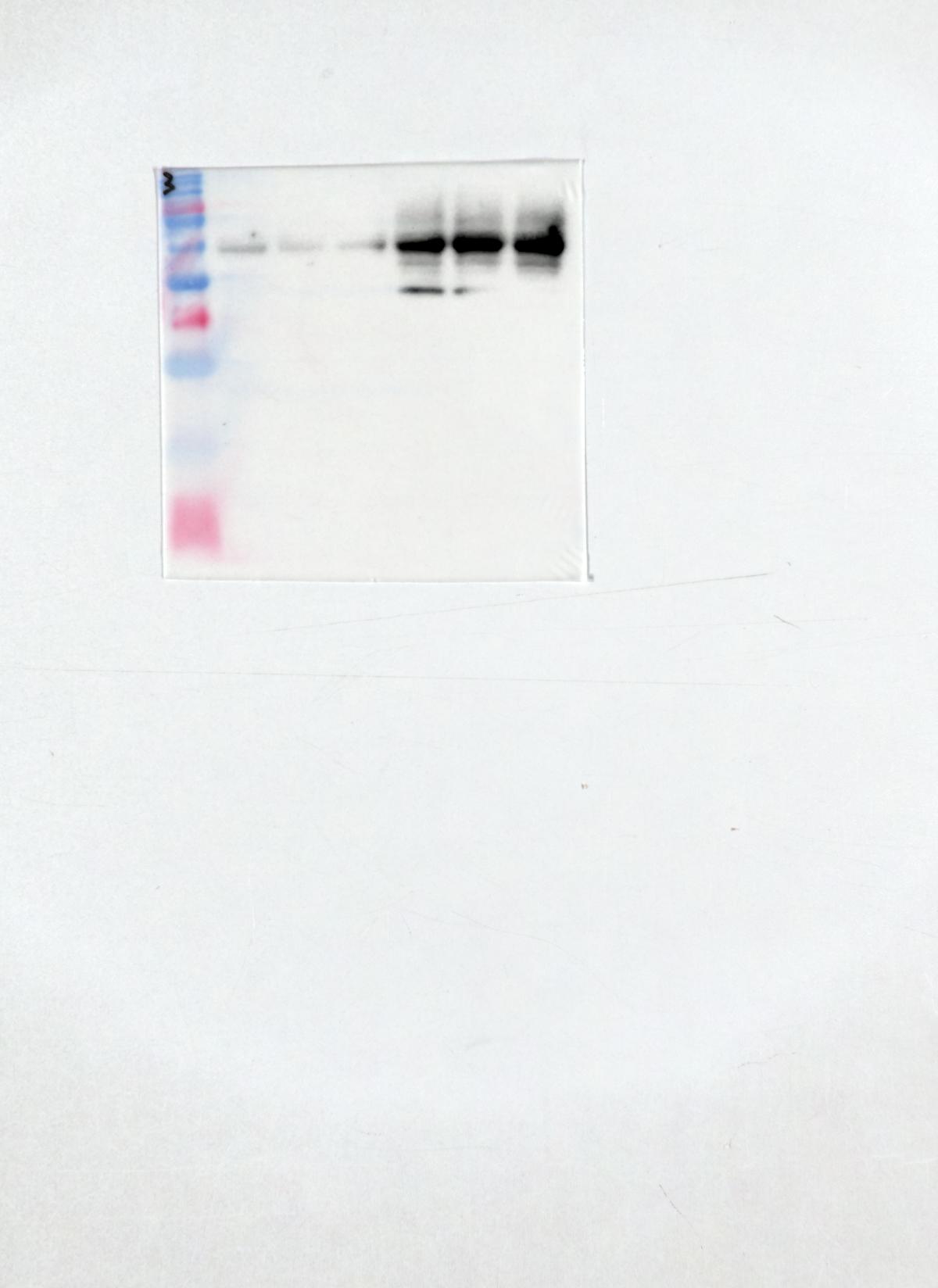

Supplement: Supplementary file 1 [file biomolecules-14-01533-s001.zip › Fig2.B PTGS2.jpg]

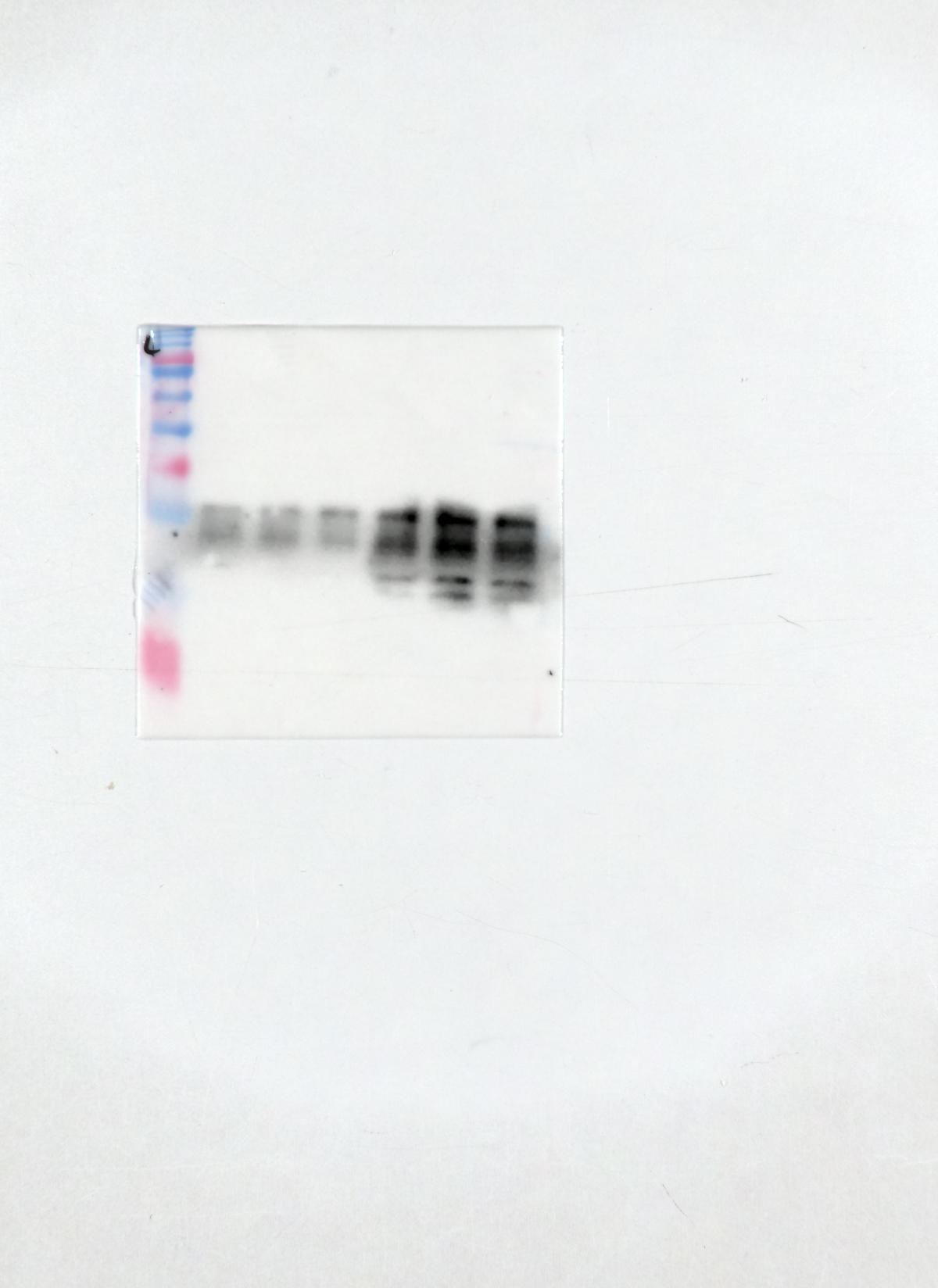

Supplement: Supplementary file 1 [file biomolecules-14-01533-s001.zip › Fig2.B TNF-a┴.jpg]

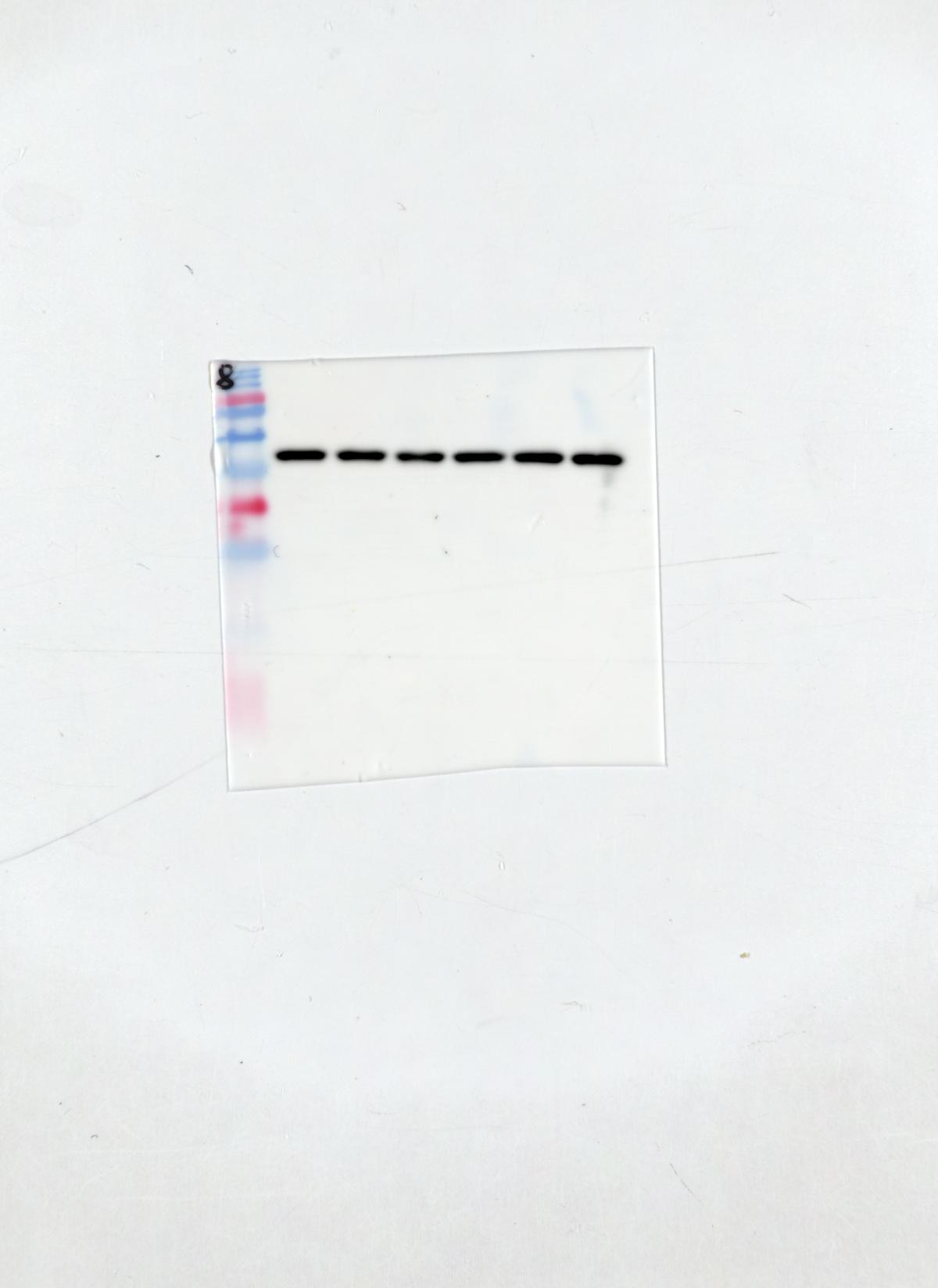

Supplement: Supplementary file 1 [file biomolecules-14-01533-s001.zip › Fig2.B a┬-actin.jpg]

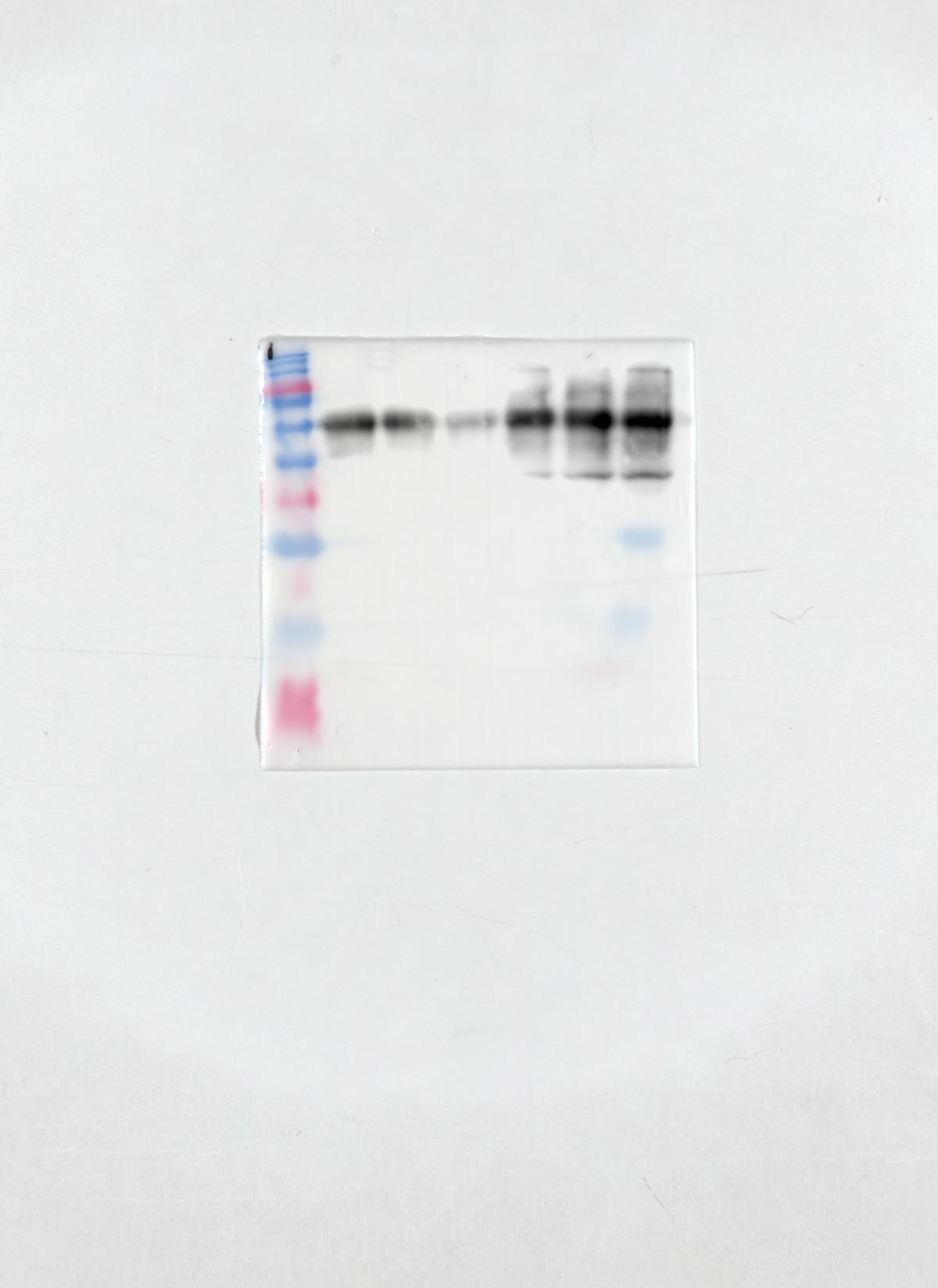

Supplement: Supplementary file 1 [file biomolecules-14-01533-s001.zip › Fig2.D ATF6.jpg]

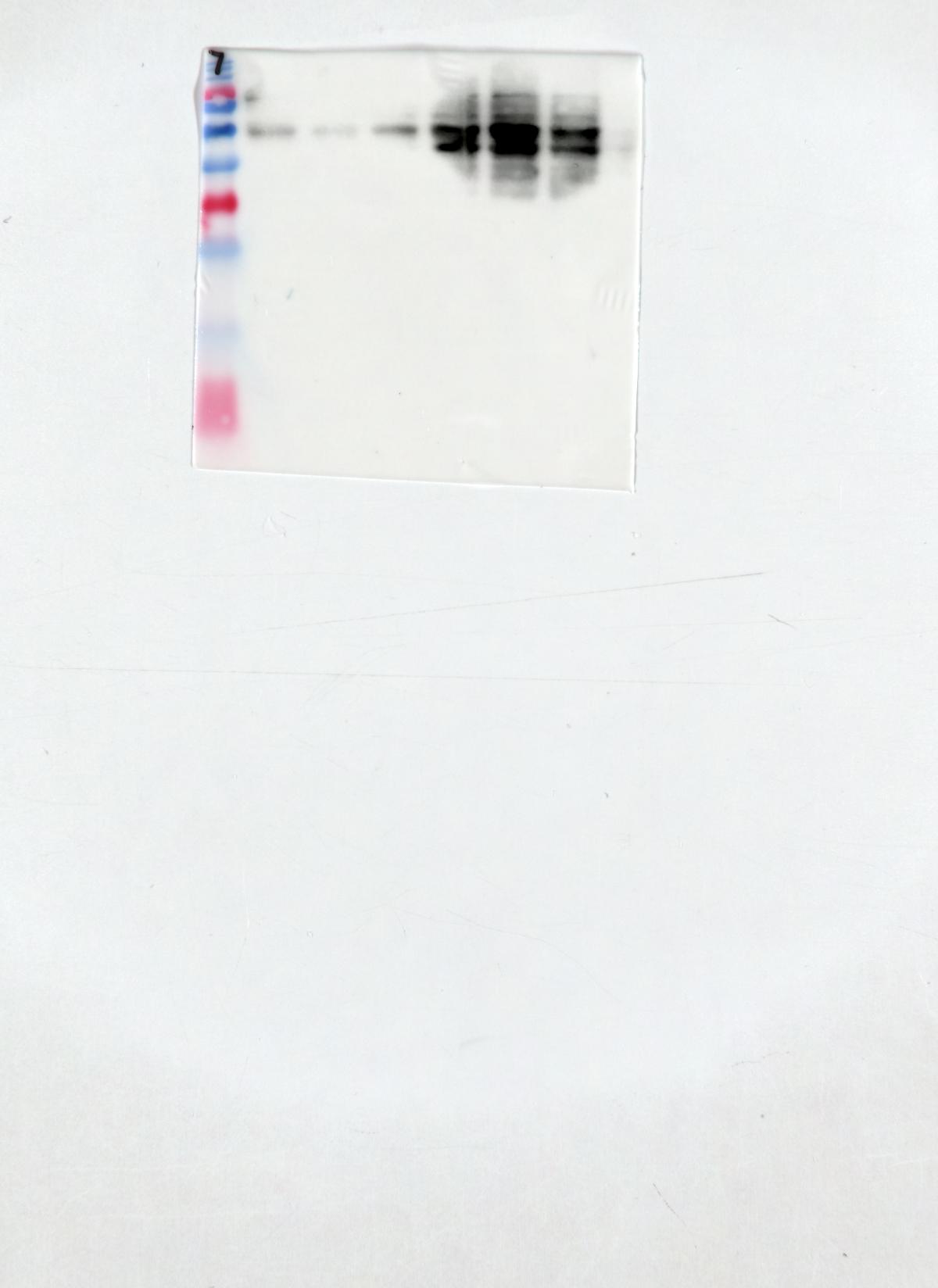

Supplement: Supplementary file 1 [file biomolecules-14-01533-s001.zip › Fig2.D GRP78.jpg]

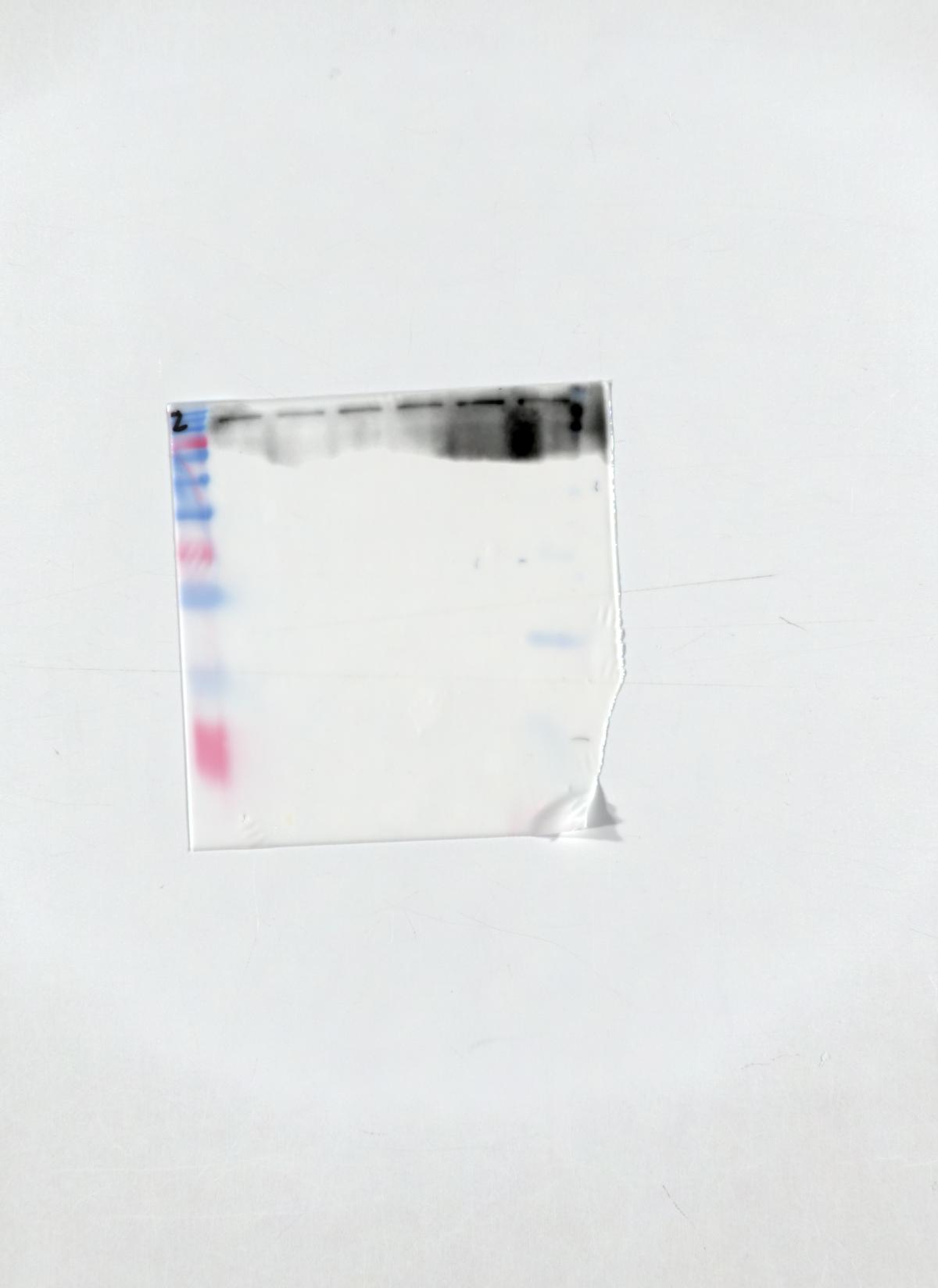

Supplement: Supplementary file 1 [file biomolecules-14-01533-s001.zip › Fig2.D PERK.jpg]

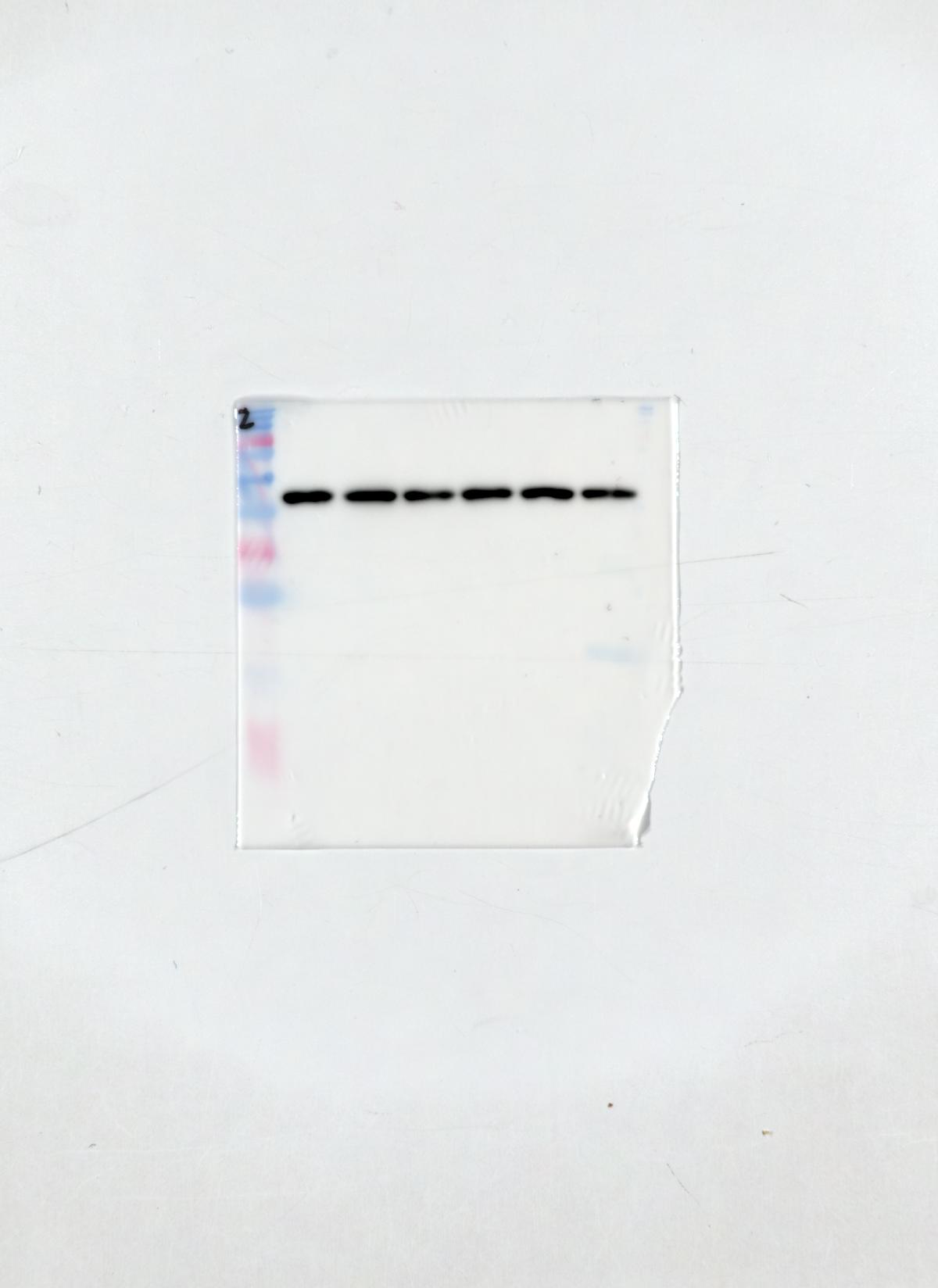

Supplement: Supplementary file 1 [file biomolecules-14-01533-s001.zip › Fig2.D a┬-actin.jpg]

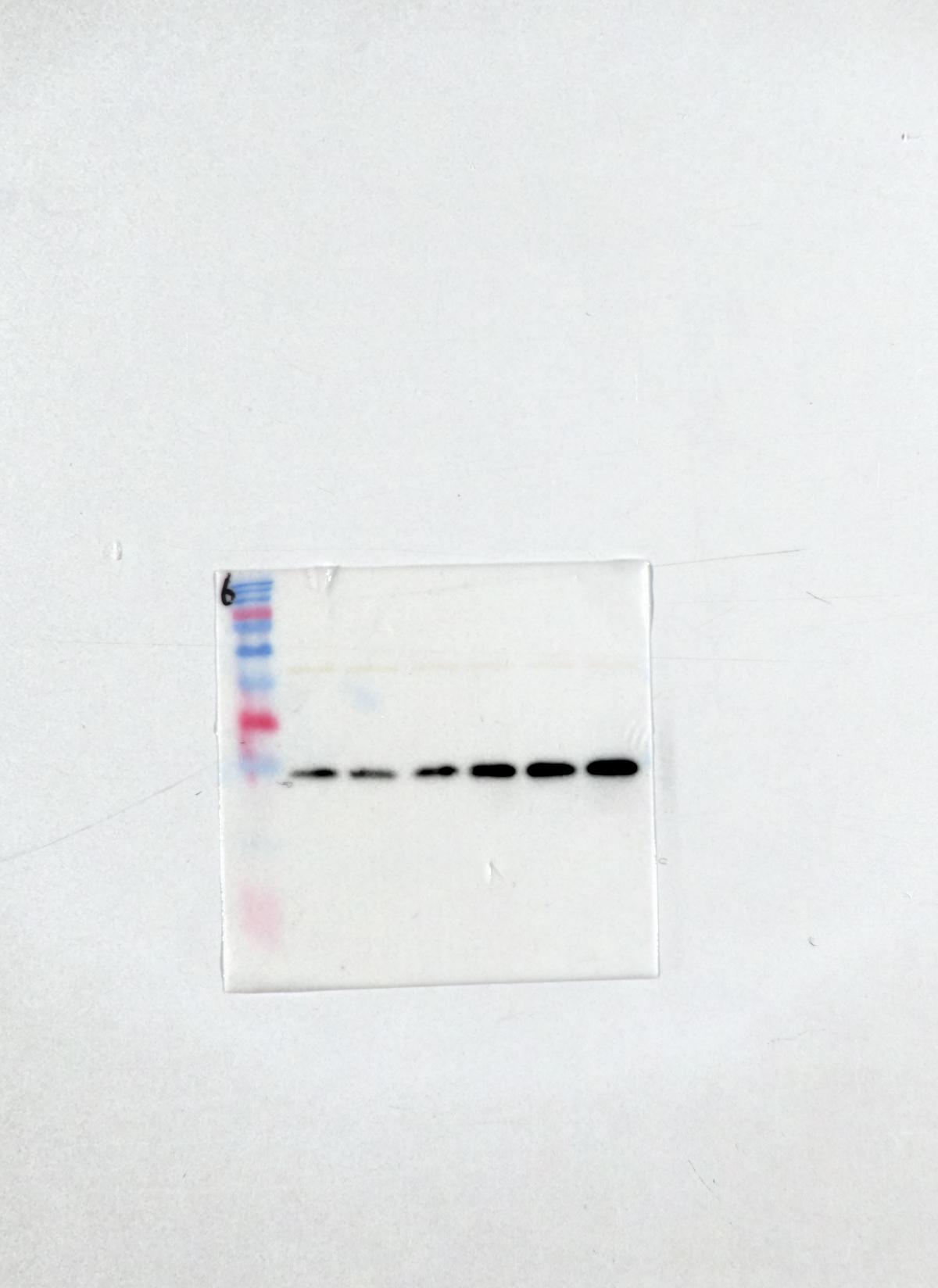

Supplement: Supplementary file 1 [file biomolecules-14-01533-s001.zip › Fig2.F BAX.jpg]

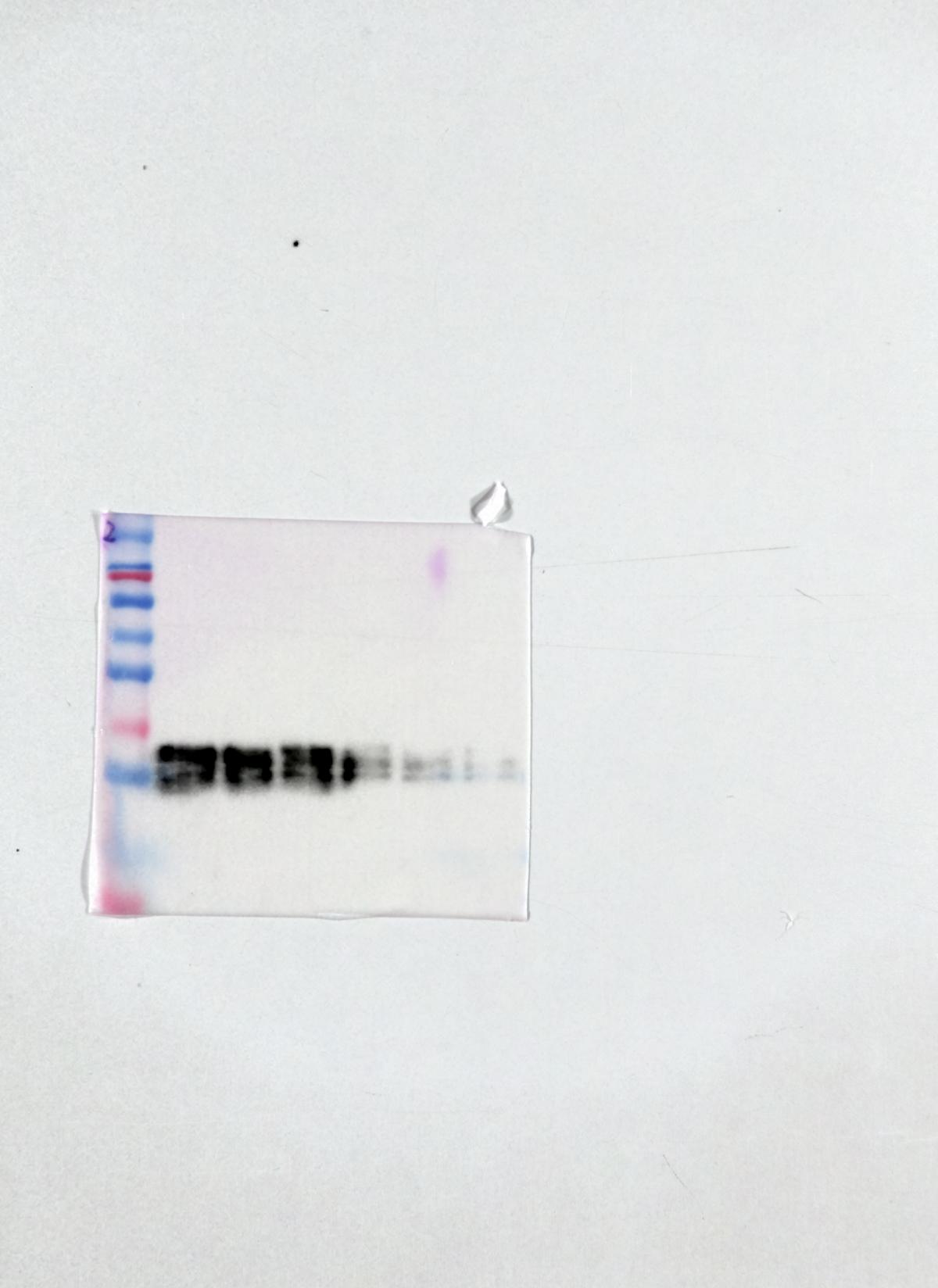

Supplement: Supplementary file 1 [file biomolecules-14-01533-s001.zip › Fig2.F Bcl-2.jpg]

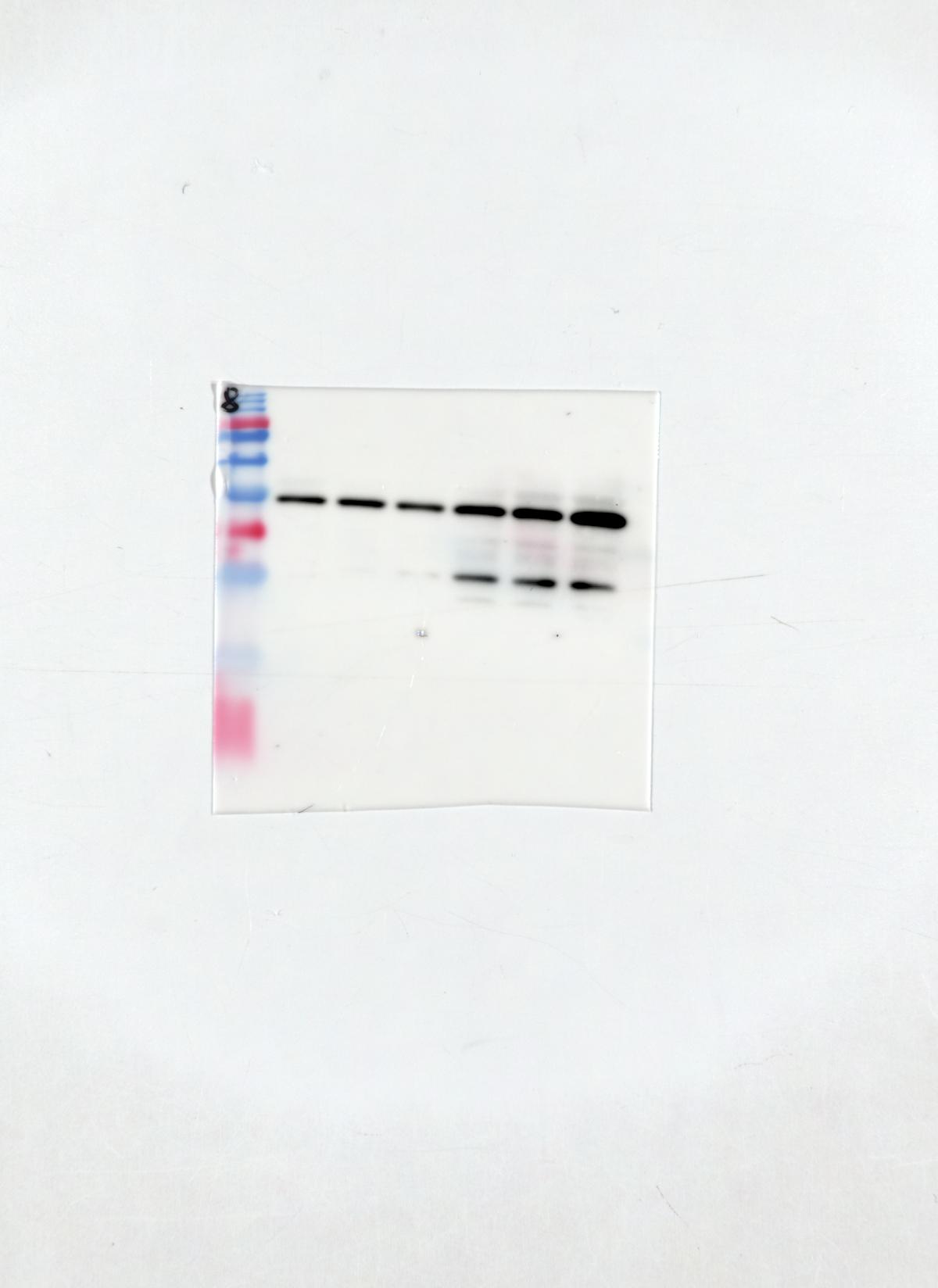

Supplement: Supplementary file 1 [file biomolecules-14-01533-s001.zip › Fig2.F Caspase3.jpg]

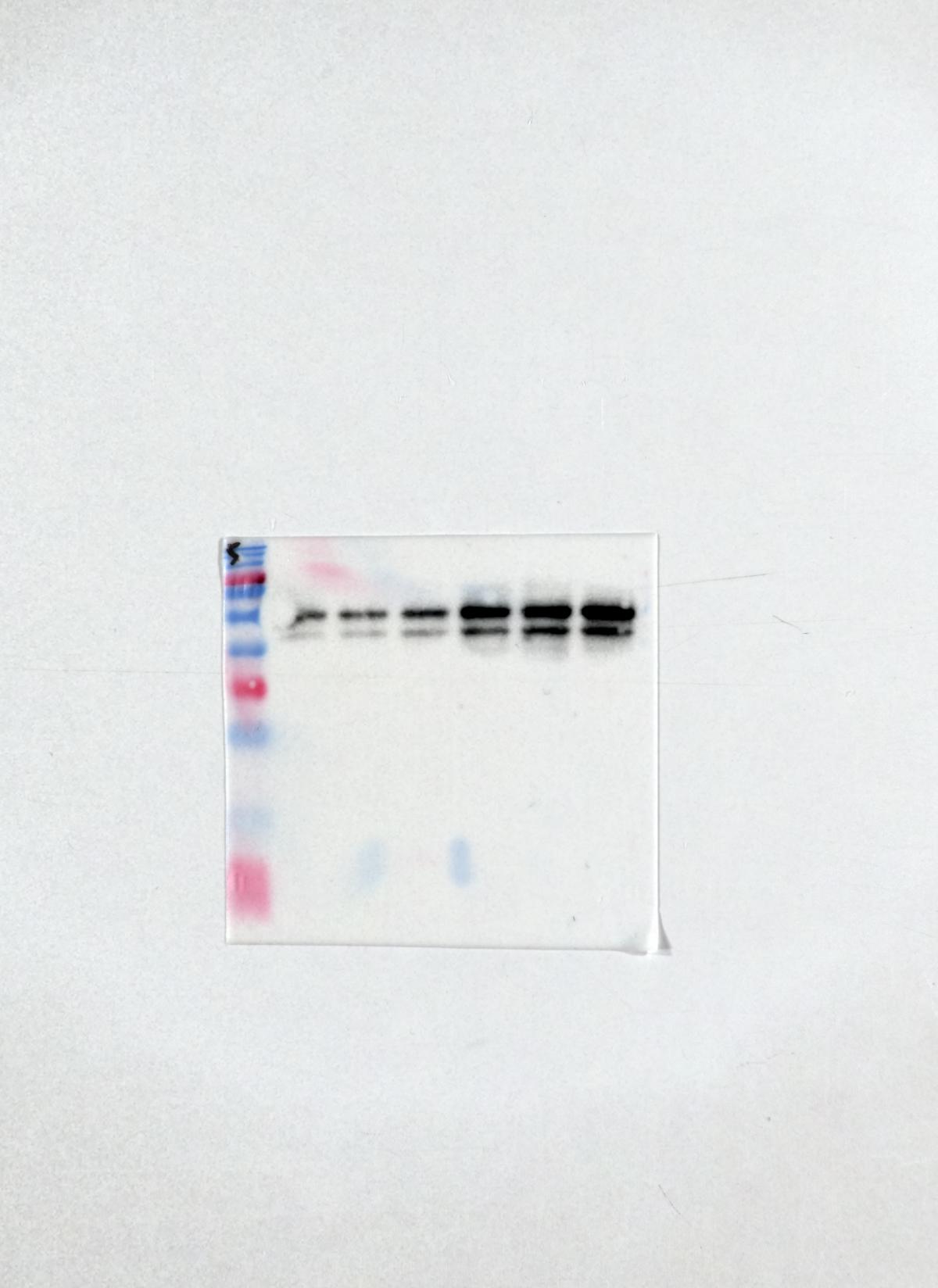

Supplement: Supplementary file 1 [file biomolecules-14-01533-s001.zip › Fig2.F Caspase8.jpg]

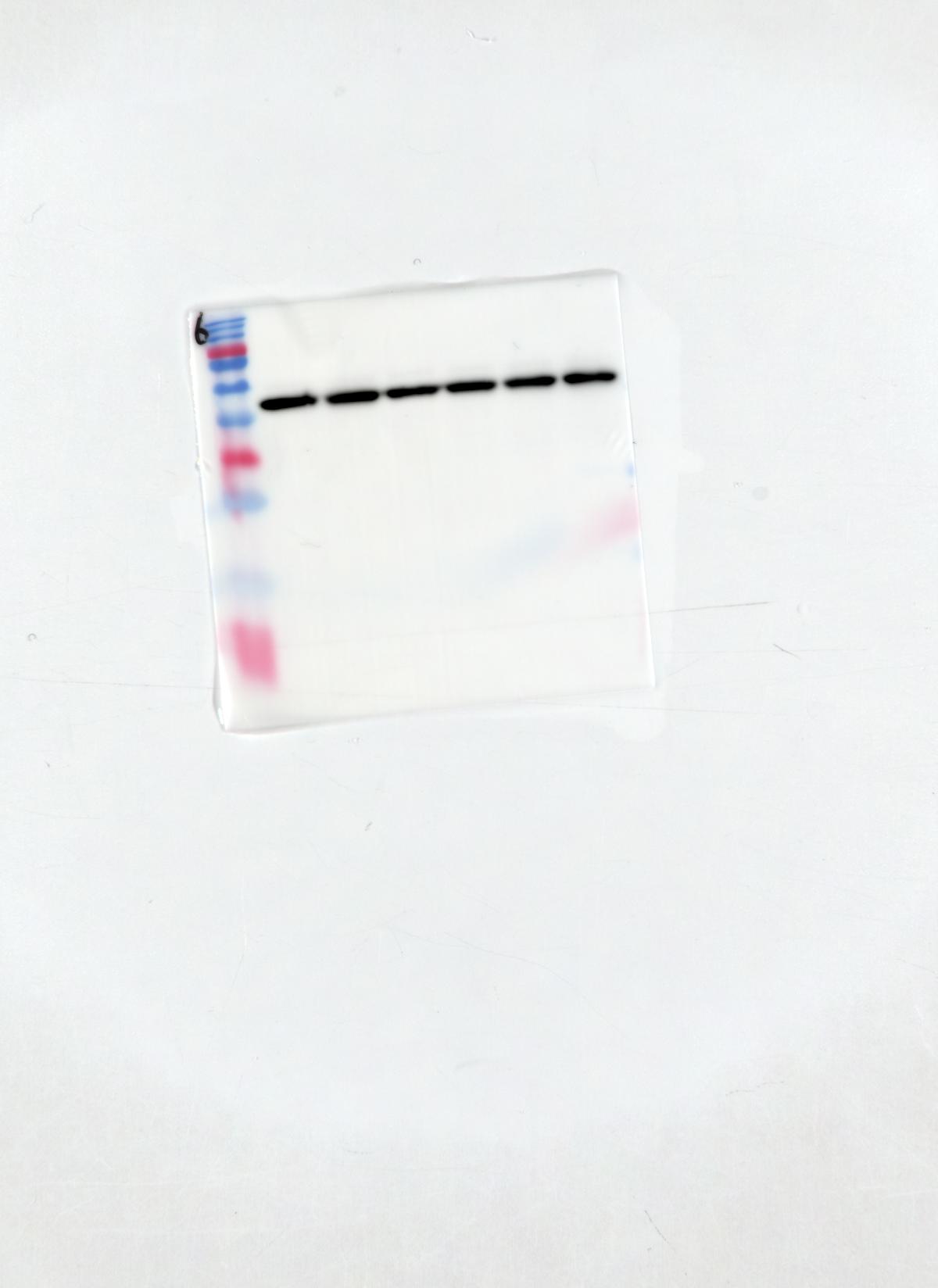

Supplement: Supplementary file 1 [file biomolecules-14-01533-s001.zip › Fig2.F a┬-actin.jpg]

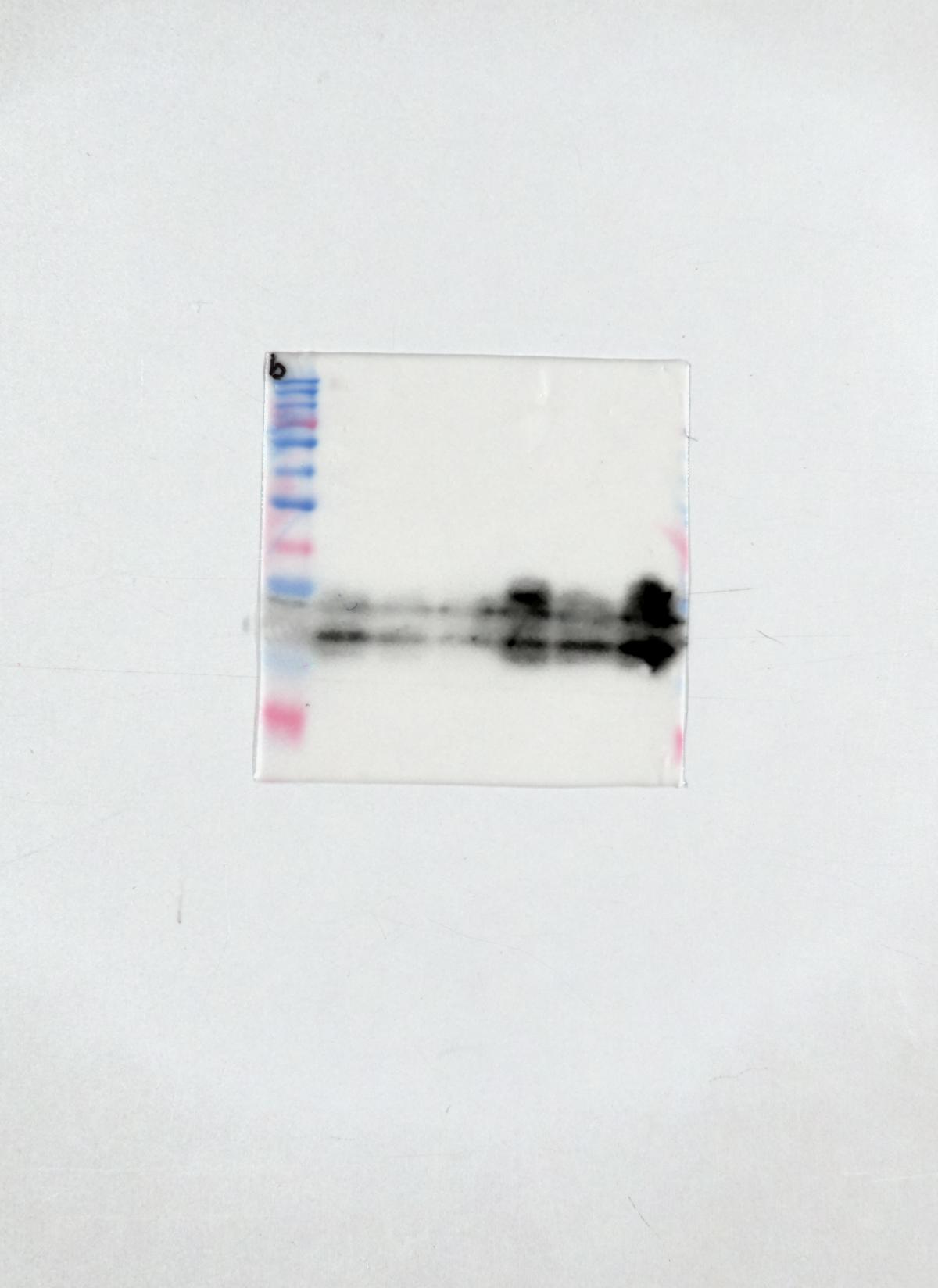

Supplement: Supplementary file 1 [file biomolecules-14-01533-s001.zip › Fig5.D IL-1a┬.jpg]

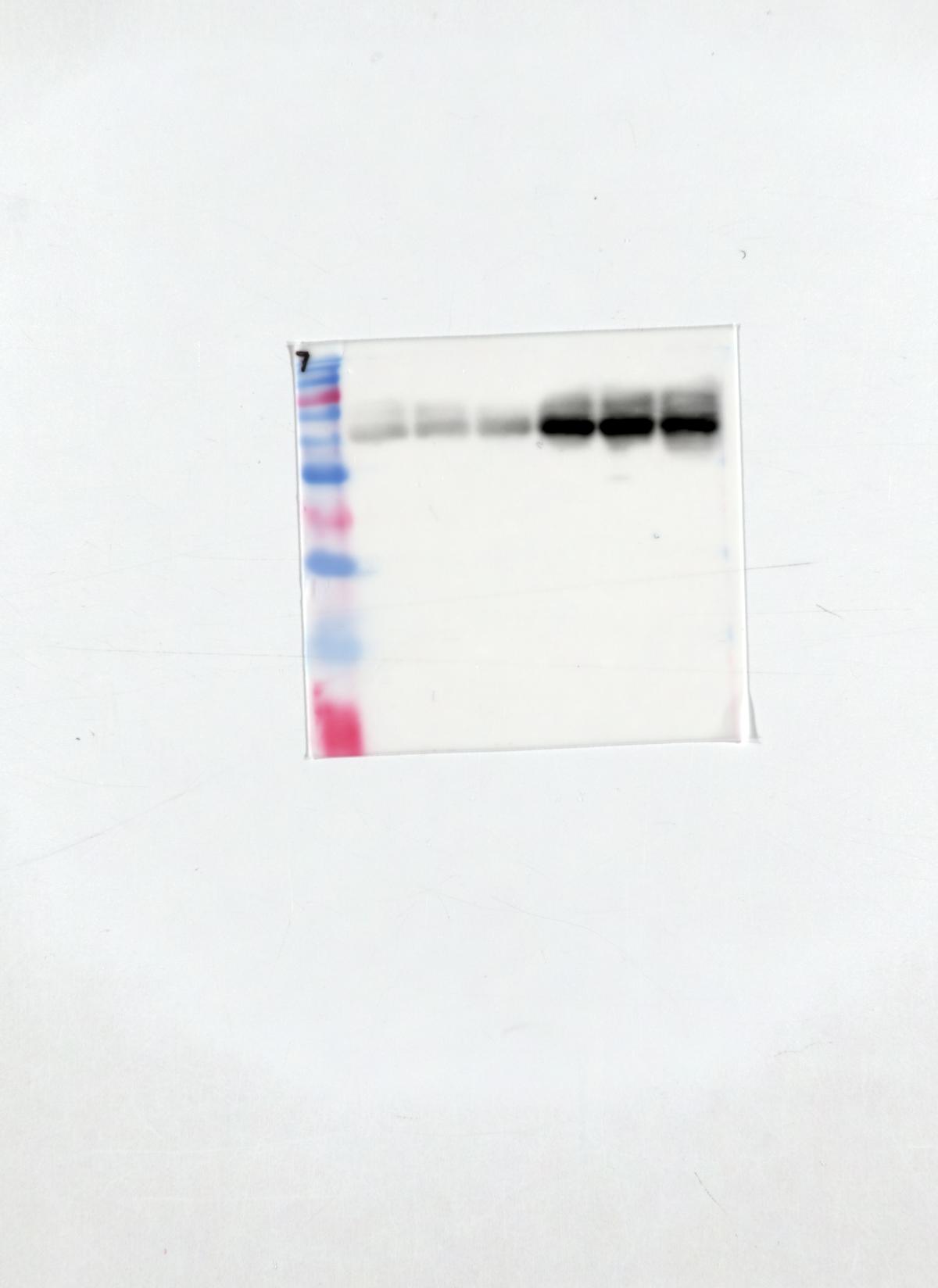

Supplement: Supplementary file 1 [file biomolecules-14-01533-s001.zip › Fig5.D PTGS2.jpg]

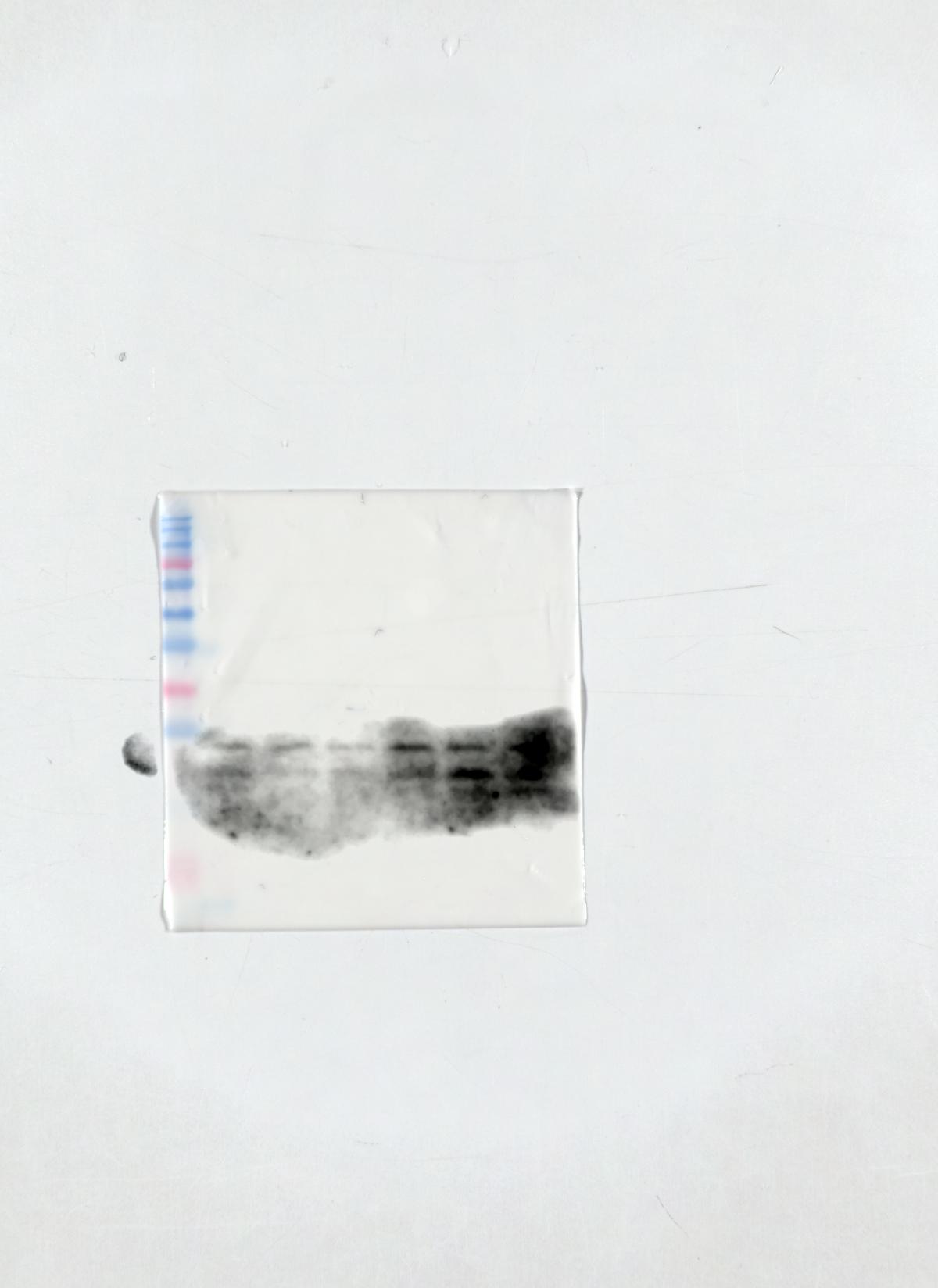

Supplement: Supplementary file 1 [file biomolecules-14-01533-s001.zip › Fig5.D TNF-a┴.jpg]

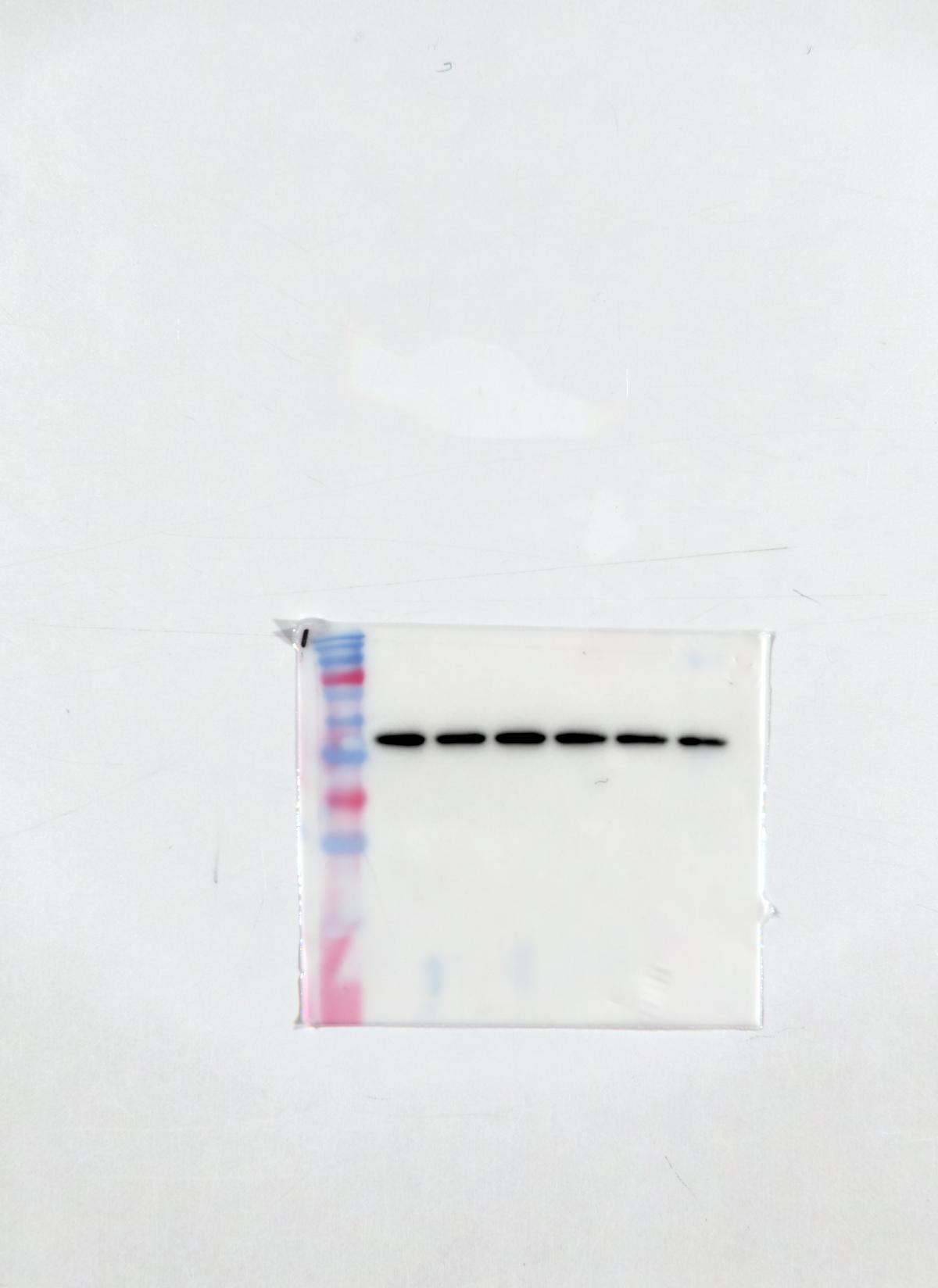

Supplement: Supplementary file 1 [file biomolecules-14-01533-s001.zip › Fig5.D a┬-actin.jpg]

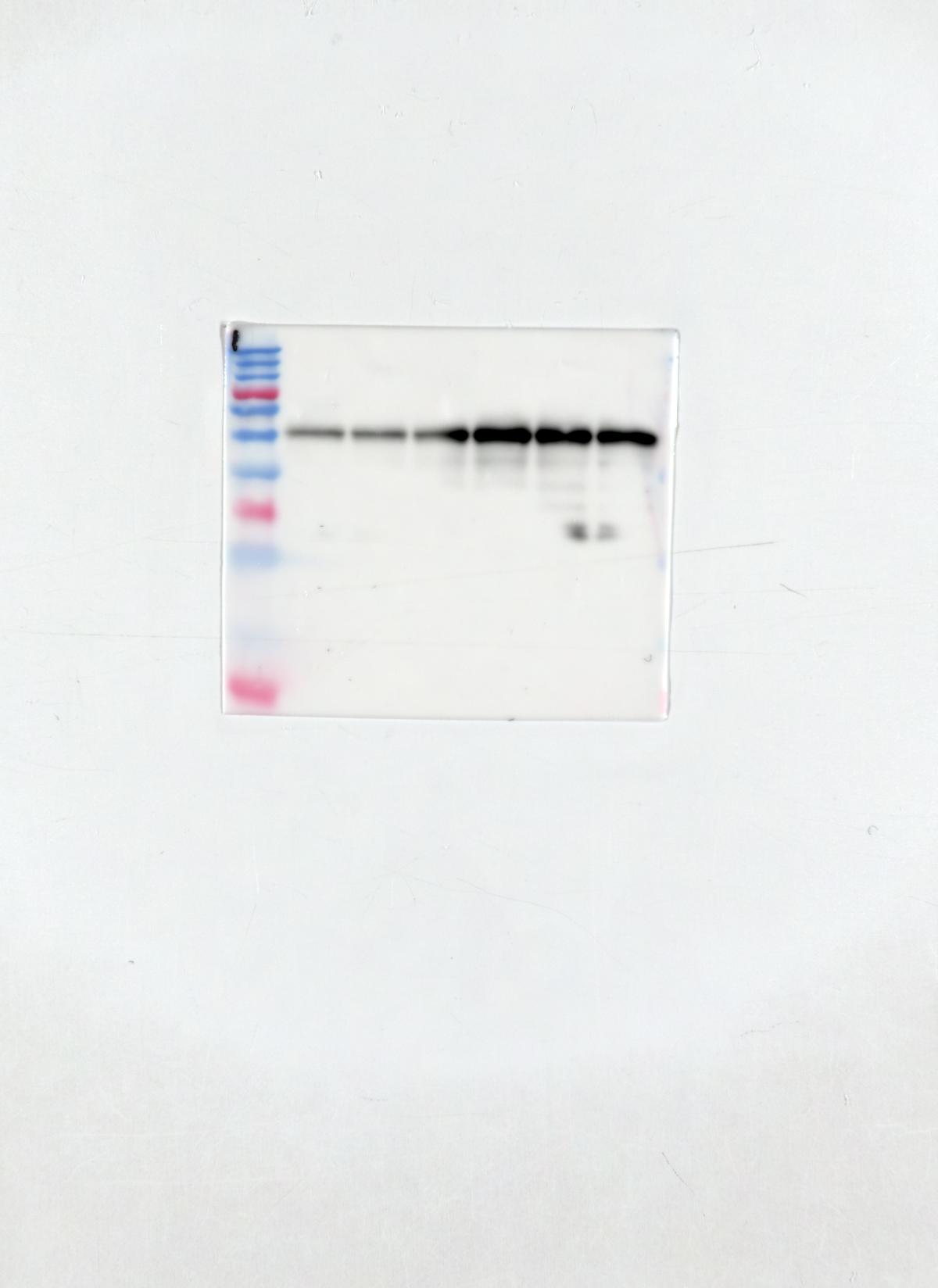

Supplement: Supplementary file 1 [file biomolecules-14-01533-s001.zip › Fig5.F ATF6.jpg]

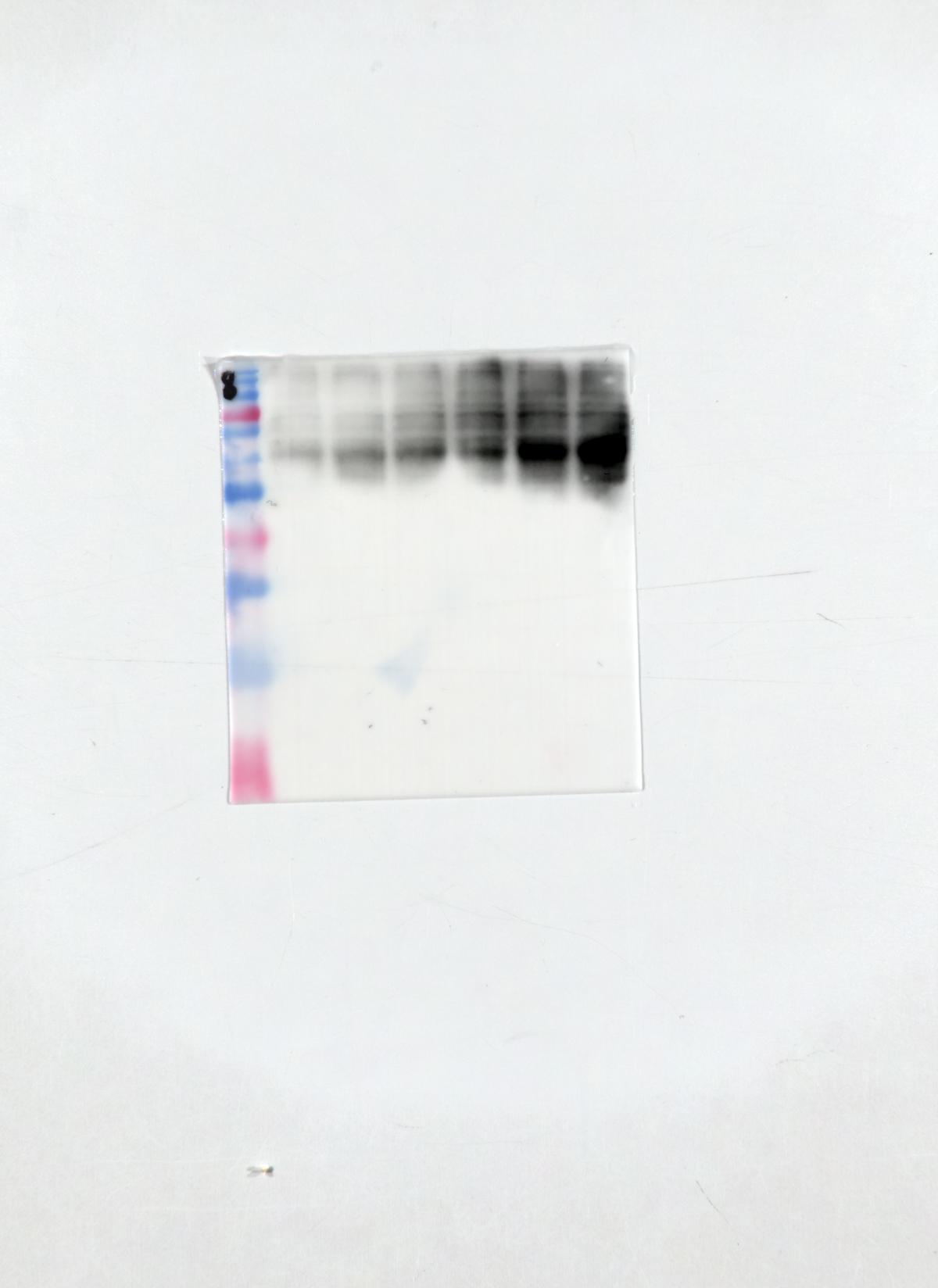

Supplement: Supplementary file 1 [file biomolecules-14-01533-s001.zip › Fig5.F GRP78.jpg]

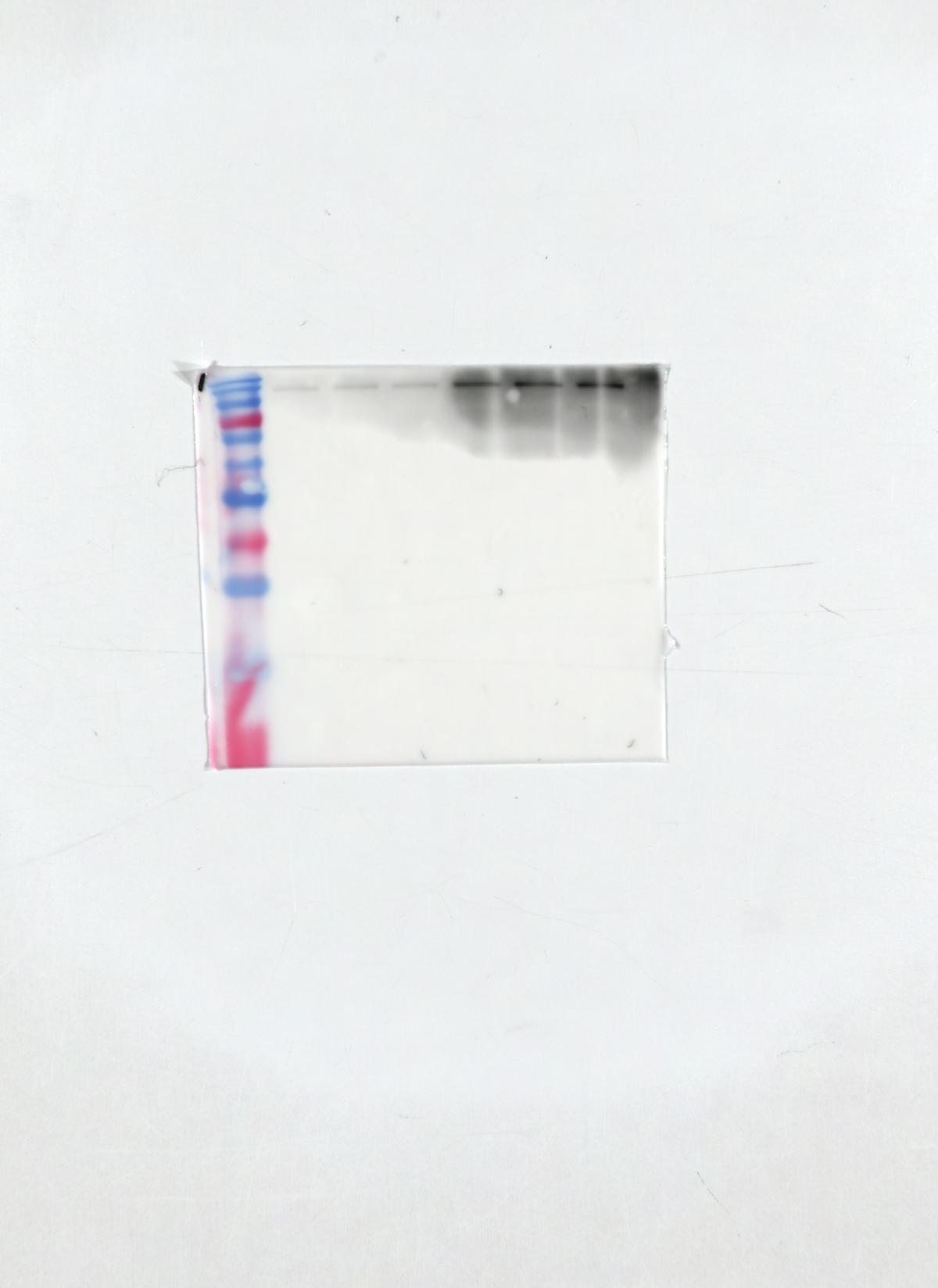

Supplement: Supplementary file 1 [file biomolecules-14-01533-s001.zip › Fig5.F PERK.jpg]

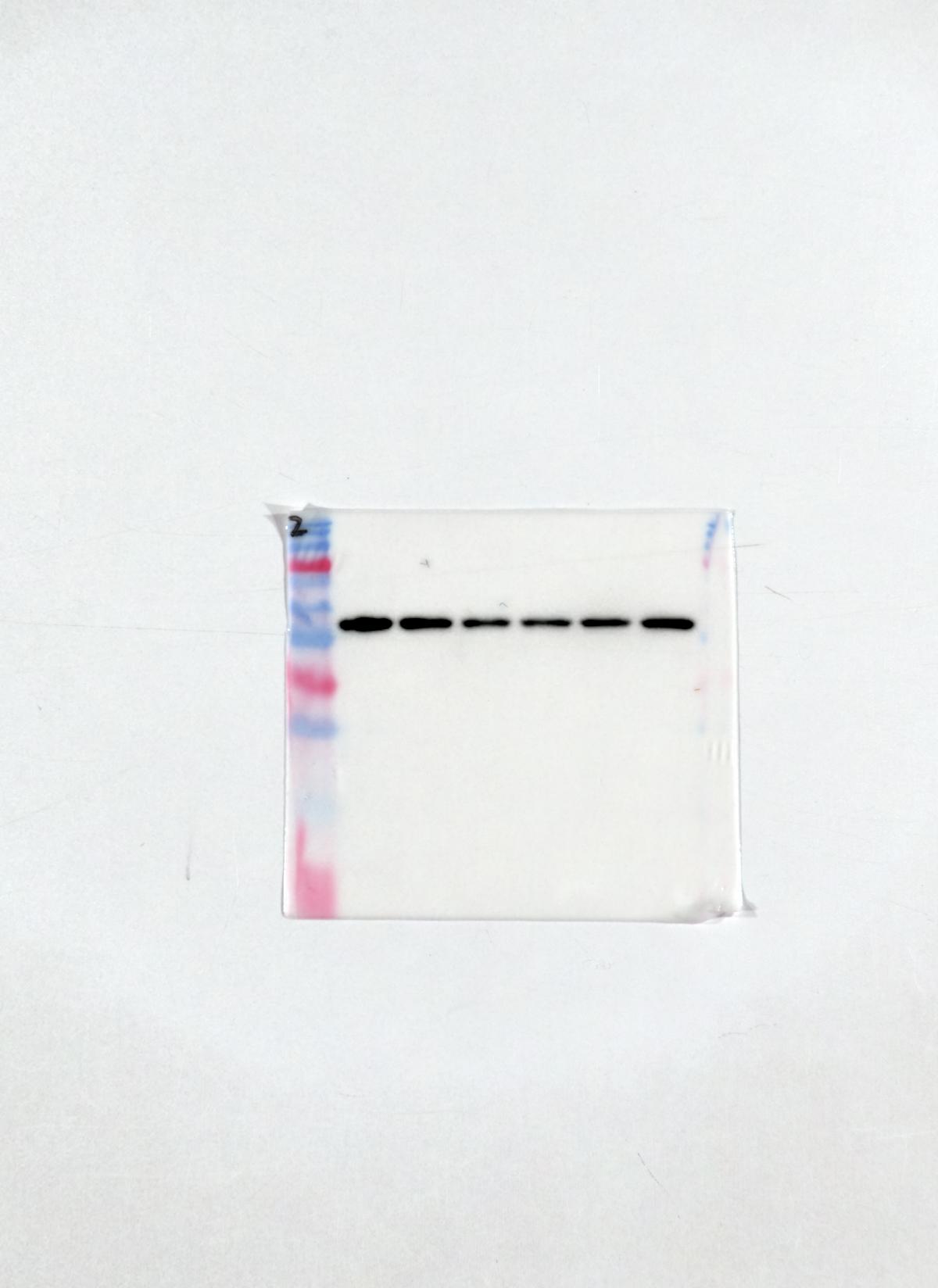

Supplement: Supplementary file 1 [file biomolecules-14-01533-s001.zip › Fig5.F a┬-actin.jpg]

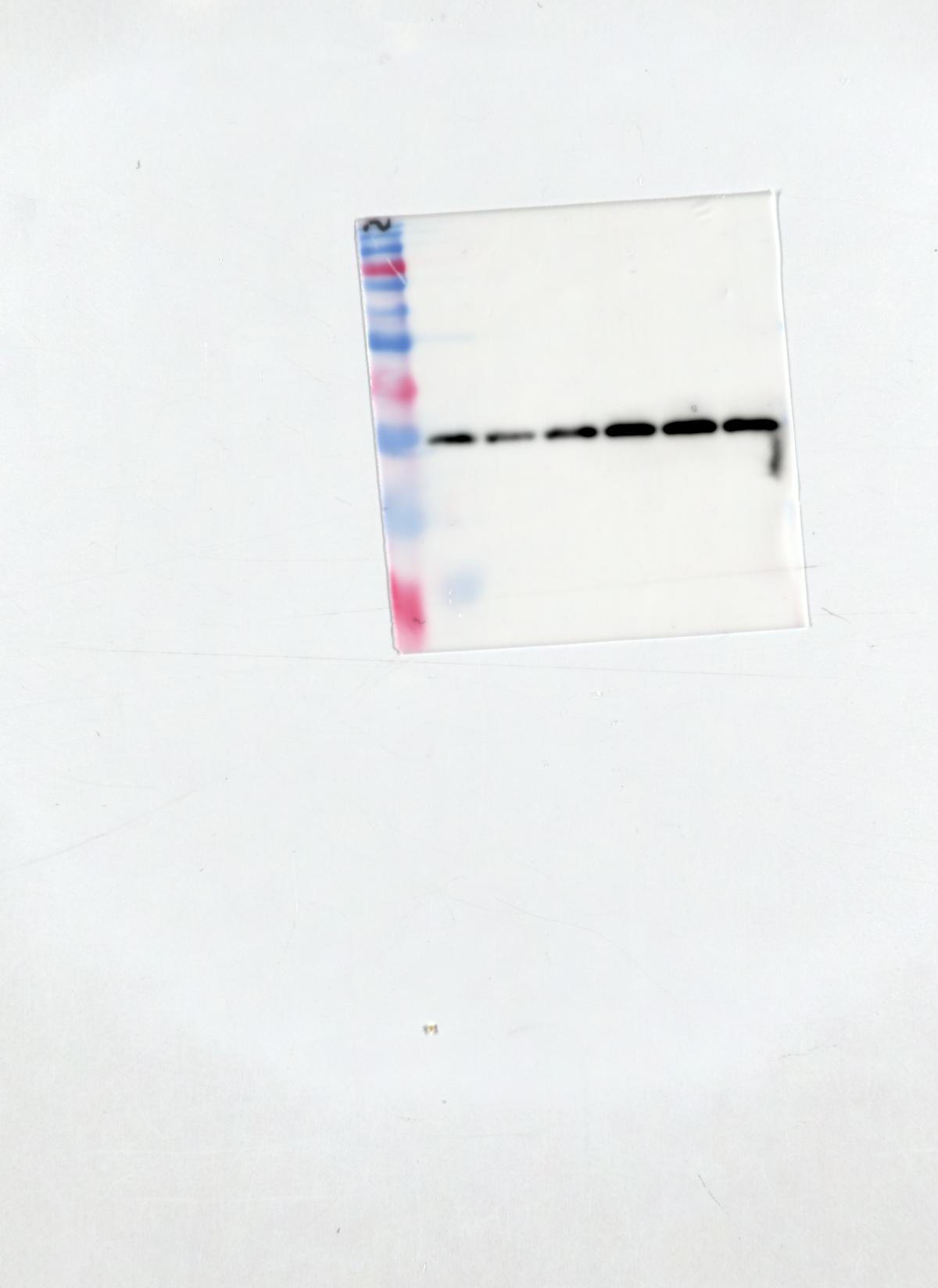

Supplement: Supplementary file 1 [file biomolecules-14-01533-s001.zip › Fig5.H BAX.jpg]

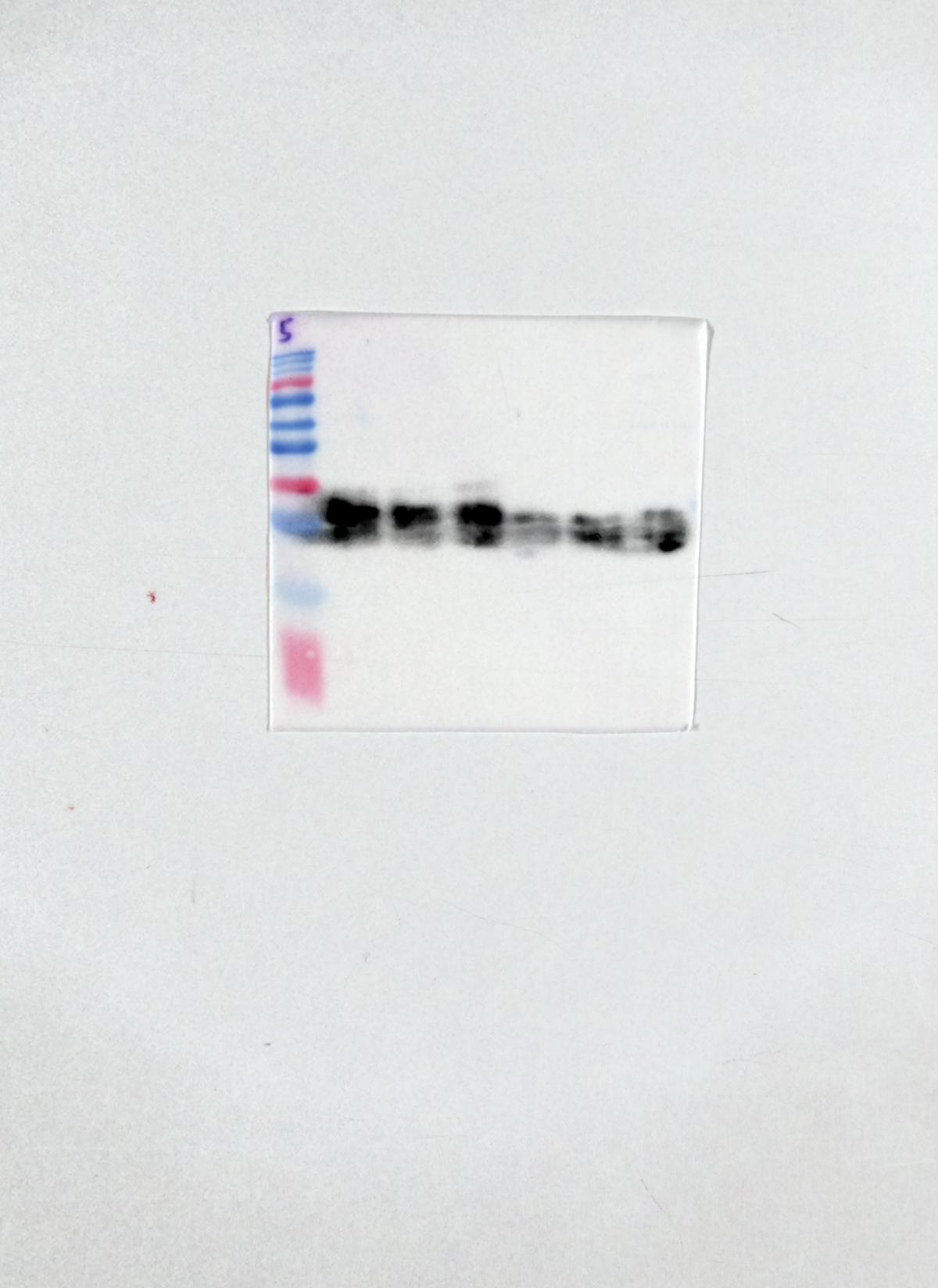

Supplement: Supplementary file 1 [file biomolecules-14-01533-s001.zip › Fig5.H Bcl-2.jpg]

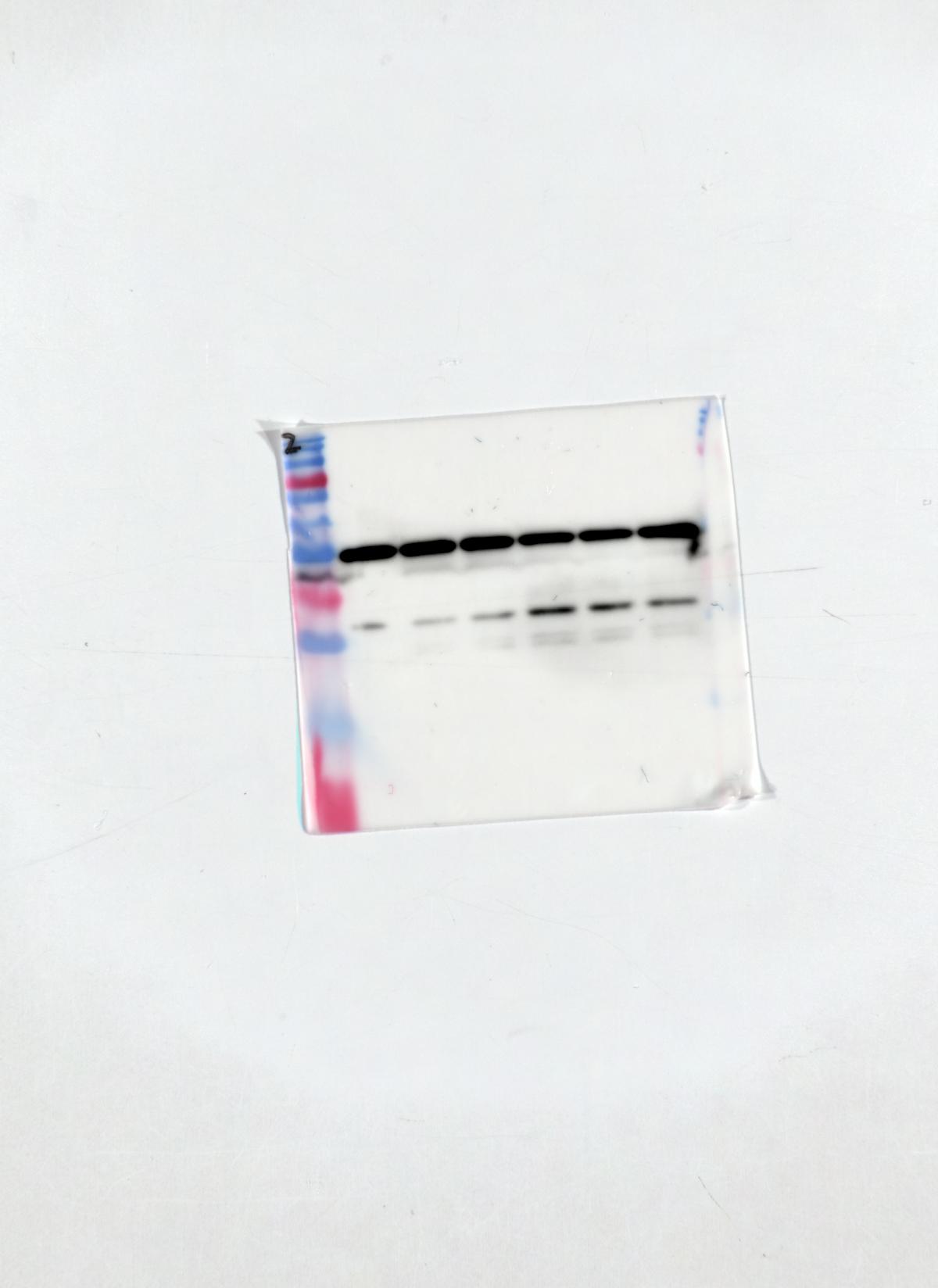

Supplement: Supplementary file 1 [file biomolecules-14-01533-s001.zip › Fig5.H Caspase3.jpg]

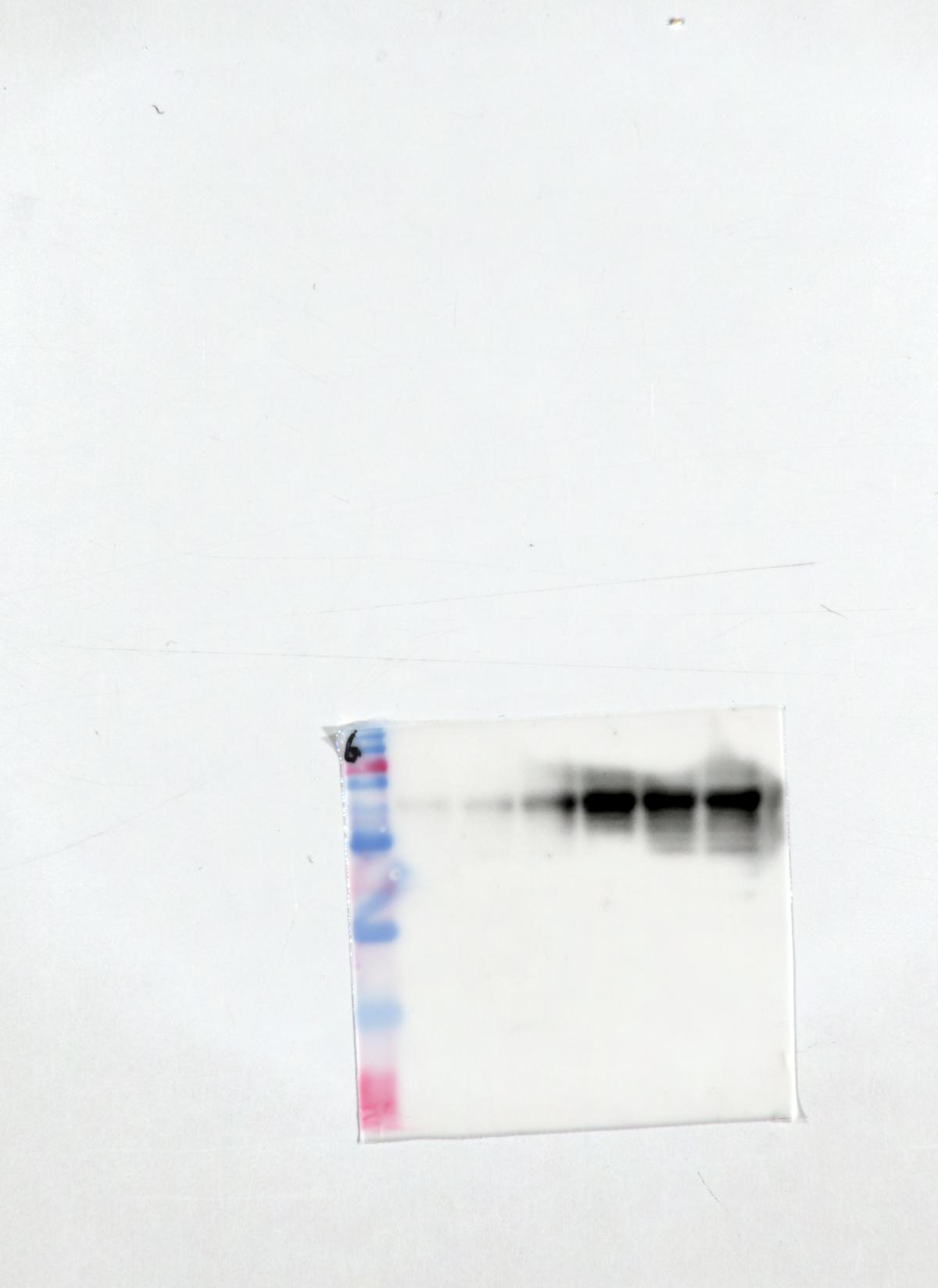

Supplement: Supplementary file 1 [file biomolecules-14-01533-s001.zip › Fig5.H Caspase8.jpg]

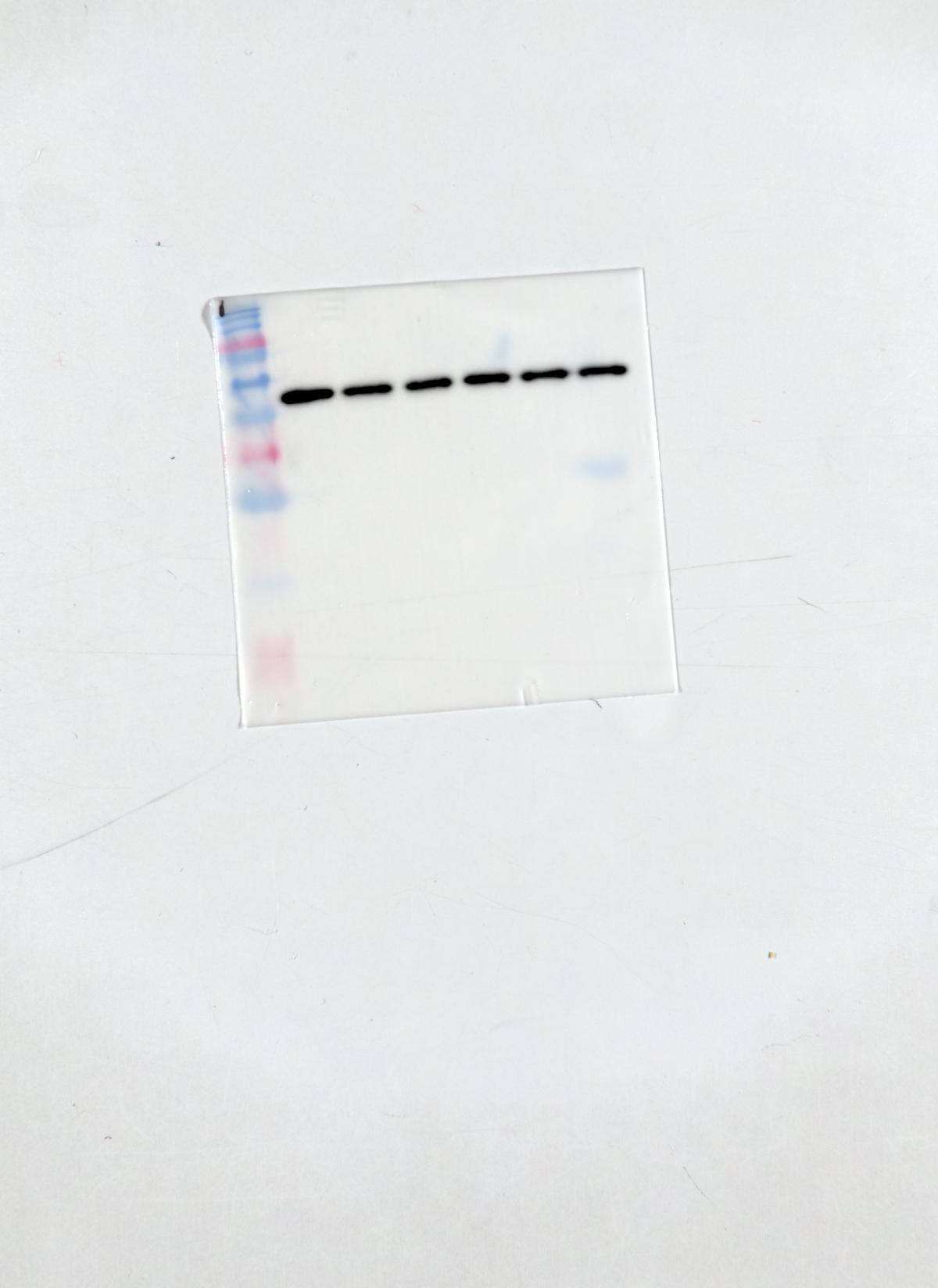

Supplement: Supplementary file 1 [file biomolecules-14-01533-s001.zip › Fig5.H a┬-actin.jpg]
